# Influence of plasma-activated compounds on melanogenesis and tyrosinase activity

**DOI:** 10.1038/srep21779

**Published:** 2016-03-02

**Authors:** Anser Ali, Zaman Ashraf, Naresh Kumar, Muhammad Rafiq, Farukh Jabeen, Ji Hoon Park, Ki Hong Choi, SeungHyun Lee, Sung-Yum Seo, Eun Ha Choi, Pankaj Attri

**Affiliations:** 1 Department of Plasma-Bio Display, Kwangwoon University, 20 Kwangwoon-gil, Nowon-gu, Seoul 139-701, Republic of Korea; 2 Plasma Bioscience Research Center, Kwangwoon University, 20 Kwangwoon-gil, Nowon-gu, Seoul 139-701, Republic of Korea; 3 Department of Biology, College of Natural Sciences, Kongju National University, Gongju 314-701, Republic of Korea; 4 Department of Chemistry, Allama Iqbal Open University, Islamabad 44000, Pakistan; 5 Department of Biochemistry & Biotechnology (Baghdad-ul-Jadeed Campus), The Islamia University of Bahawalpur 63100, Pakistan; 6 Florida Center of Heterocyclic Compounds, University of Florida, Gainesville, Fl, 32601, USA; 7 Center for Computationally Assisted Science and Technology, North Dakota State University, Fargo, ND, 58102, USA; 8 Department of Electrical and Biological Physics, Kwangwoon University, 20 Kwangwoon-gil, Nowon-gu, Seoul 139-701, Republic of Korea; 9 Graduate School of Information Science and Electrical Engineering, Kyushu University, Fukuoka 819-0395, Japan

## Abstract

Many organic chemists around the world synthesize medicinal compounds or extract multiple compounds from plants in order to increase the activity and quality of medicines. In this work, we synthesized new eugenol derivatives (ED) and then treated them with an N_2_ feeding gas atmospheric pressure plasma jet (APPJ) to increase their utility. We studied the tyrosinase-inhibition activity (activity test) and structural changes (circular dichroism) of tyrosinase with ED and plasma activated eugenol derivatives (PAED) in a cell-free environment. Later, we used docking studies to determine the possible interaction sites of ED and PAED compounds with tyrosinase enzyme. Moreover, we studied the possible effect of ED and PAED on melanin synthesis and its mechanism in melanoma (B16F10) cells. Additionally, we investigated the structural changes that occurred in activated ED after plasma treatment using nuclear magnetic resonance (NMR). Hence, this study provides a new perspective on PAED for the field of plasma medicine.

Melanogenesis is the process of melanin synthesis in melanosomes by melanocytes^1^. During this process, a series of changes occurs in melanocytes such as enhanced cell size and number, increased melanosomes with enhanced melanin production, increased melanocyte dendricity for increased transport of melanosomes to the neighboring keratinocytes, and increased multiplication of keratinocytes resulting in thickening of the stratum corneum and skin epidermis^2^. Many signaling pathways help to produce melanin in cells, amongst which the cyclic adenosine monophosphate (cAMP) pathway is the most studied for melanin production^3^,^4^. The cAMP stimulation results in the up-regulation of microphthalmia-associated transcription factor (MITF), tyrosinase, and tyrosinase-related proteins (Tyrp1 and Tyrp 2)^5^,^6^,^7^. The stimulation of cAMP can be affected by several factors such as adrenocorticotropic hormone (ACTH), prostaglandin E2 (PGE2), and alpha-melanocyte stimulating hormone (α-MSH)^3^,^4^,^8^,^9^. The increase in melanogenesis without affecting the skin is very important, because it can protect the skin from diseases such as cancer^10^ and vitiligo^11^. The tanning caused by UV irradiation can result in DNA damage, gene mutation, cancer development, impairment of the immune system or photoaging^2^. Hence, many researchers have examined natural or synthesized compounds such as scoparone to regulate melanin synthesis^2^,^12^,^13^,^14^. However, a photoprotective tanning agent or a more efficient compound is needed, one that increases melanogenesis to protect the skin from damage but does not affect metabolism.

Melanocytes are also localized in hair follicles that provide pigment to hair and skin. The melanocytes repeatedly proliferate and differentiate in hair follicles for pigmentation. Fully mature melanocytes produce melanin pigments in an organelle called melanosomes, which are then transferred to neighboring keratinocytes that create the hair shaft, whereby the growing hair becomes pigmented during each hair cycle^15^,^16^. The follicular repigmentation of depigmented vitiligo skin after narrow band UVB treatment has been reported^11^,^17^,^18^. The repigmentation usually starts at the openings of hair follicles, then enlarges and coalesces to cover the entire skin area depigmented by a pigmentary skin disorder such as vitiligo^11^. This phenomenon is known as ‘follicular repigmentation’ and is characterized by the repigmentation of an un-pigmented skin area starting from the hair follicle^17^,^18^. Therefore, melanogenic compounds may be helpful in activating the melanocytes to produce the melanin pigment necessary to maintain the hair color for long periods and to re-pigment the unpigmented skin area caused by pigmentary skin disorders such as vitiligo.

The High energy electrons present in NTP generates free radicals, including reactive oxygen species (ROS) and reactive nitrogen species (RNS) that play an important role in plasma medicine^19^,^20^,^21^,^22^,^23^,^24^,^25^,^26^. Studies have reported NTP as a novel and desirable technology for food and bio decontamination^27^,^28^. However, a recent study by Kim *et al.*^29^ showed that plasma can enhance the biological activity of natural material naringin. This material can show increased tyrosinase inhibition and anti-microbial activities after plasma treatment^29^. Considering this, we synthesized new eugenol derivatives (ED) and then treated them with N_2_ feeding gas atmospheric pressure plasma jet (APPJ) and also investigated the structure of the most active ED (4b and 6a) after the plasma treatment by NMR. Using ED and plasma-activated eugenol derivatives (PAED) compounds, we have studied their tyrosinase-inhibition activity and structural changes of the tyrosinase enzyme by activity test and circular dichroism (CD), respectively. Further, using docking studies we checked the interaction sites of the ED with tyrosinase enzyme. We also studied melanin synthesis in melanoma (B16F10) cells by these compounds. Additionally, we investigated the mechanism of melanin synthesis using ED and PAED compounds on B16F10 cells by checking the level of cyclic adenosine monophosphate (cAMP) and intracellular ROS production, and western blotting analysis for microphthalmia-associated transcription factor (MITF), tyrosinase and tyrosinase-related protein-1 (TRP1). Hence, this study provided a new perspective on plasma activated compounds for the plasma medicine field.

## Results

### Synthesis of eugenol derivatives (ED) and study of plasma action on ED

**Figure 1:**
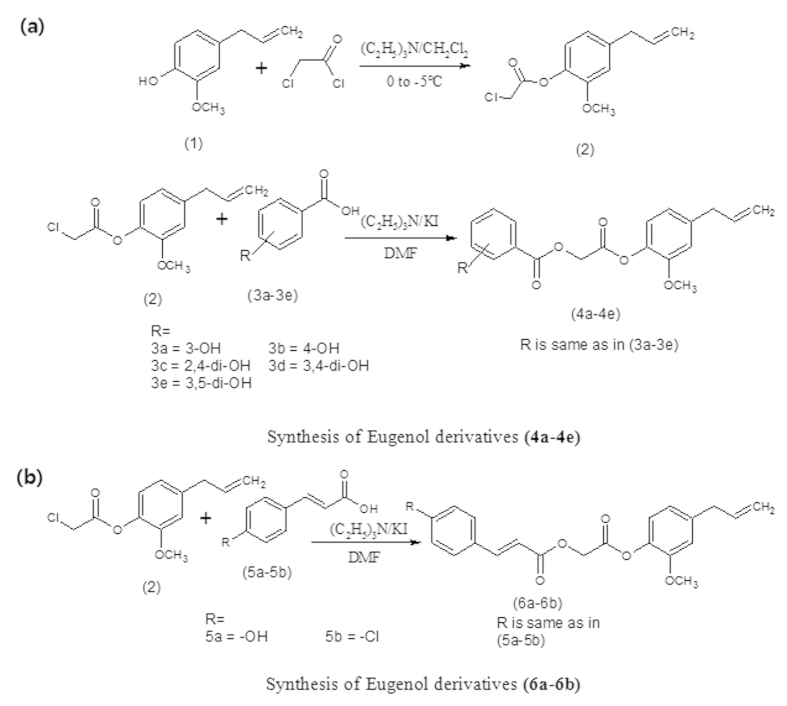
Synthesis scheme of eugenol derivatives (**a**) (4a-4e) and (**b**) (6a-6b).

**Figure 2:**
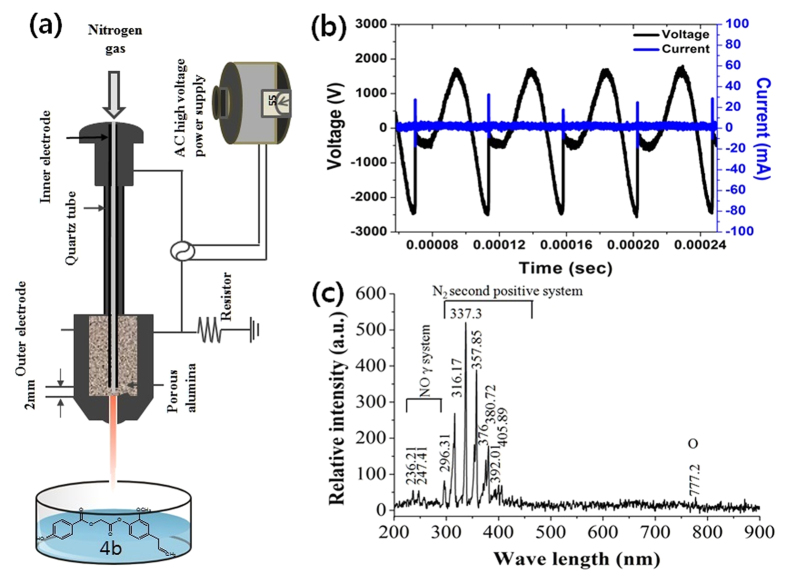
Atmospheric pressure plasma jet (APPJ) and its characteristics. (**a**) Schematic diagram of APPJ and its treatment to the test compounds. (**b**) Voltage and current graph for APPJ. (**c**) Optical emission spectrum detected from the plasma source using HR4000 spectrometer.

**Figure 3:**
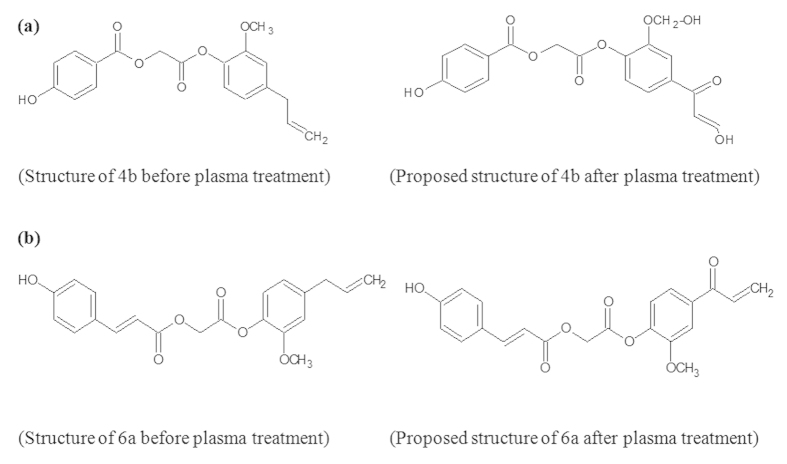
Proposed structures for (**a**) 4b, and (**b**) 6a compounds after the plasma treatment.

In this study we synthesized and characterized the ED [2-[2-methoxy-4-(prop-2-en-1-yl)phenoxy]-2-oxoethyl 3-hydroxybenzoate (4a), 2-[2-methoxy-4-(prop-2-en-1-yl)phenoxy]-2-oxoethyl 4-hydroxybenzoate (4b), 2-[2-methoxy-4-(prop-2-en-1-yl)phenoxy]-2-oxoethyl 2,4-dihydroxybenzoate (4c), 2-[2-methoxy-4-(prop-2-en-1-yl)phenoxy]-2-oxoethyl 3,4-dihydroxybenzoate (4d), 2-[2-methoxy-4-(prop-2-en-1-yl)phenoxy]-2-oxoethyl 3,5-dihydroxybenzoate (4e), 2-[2-methoxy-4-(prop-2-en-1-yl)phenoxy]-2-oxoethyl(2E)-3-(4-hydroxyphenyl)prop-2-enoate (6a), and 2-[2-methoxy-4-(prop-2-en-1-yl)phenoxy]-2-oxoethyl(2E)-3-(4-chloroyphenyl)prop-2-enoate (6b)] as shown in Fig. 1, using the method described in the material method section. Latter, we have treated above mention ED compounds with atmospheric pressure plasma jet (APPJ) using N_2_ as feeding gas, as shown in Fig. 2. After the plasma treatment, we investiagted the structural changes of the most active ED (4b and 6a) compounds in our present study using NMR. The possible proposed structure of 4b and 6a compounds after plasma treatment, are shown in Fig. 3(a,b). The NMR spectra showed that the total numbers of protons decreased and compounds (4b and 6a) oxidized after plasma treatment (Figure S2). The ripples over the NMR peaks indicate the integration of signals either for one, two or more protons. The structural changes occur at two functionalities in compound 4b after plasma treatment which can be verified by observing the changes in the integration of the signal for –OCH_3_ group at 3.77 ppm and terminal -CH = CH_2_ group at 5.17 ppm (Figure S2(a), and (Fig. 3(a)). Before plasma treatment the signals at 3.77 ppm and 5.17 ppm are for three protons and two protons respectively which were later decreased to two protons and one protons after plasma treatment, respectively (Figure S2(a)). In case of compound 6a, the benzylic methylene (–CH_2_) protons were reduced from two to zero as denoted by disappear of signal at 3.18ppm and the methylene carbon is oxidized into carbonyl group after plasma treatment as depicted in Figure S2(b) and (Fig. 3(b)). The overall structure change in 4b and 6a ED compounds is due to radical generated by APPJ. If we see the optical emission spectra (OES) in Figures S2c and S1, we observed emission lines from a molecular NO β, γ system, superoxide anion (O_2_^2−^), hydroxyl (OH) radical, N_2_ (C–B) second positive system and atomic oxygen. The generation of OH radical, superoxide and atomic oxygen are the main species generated during plasma treatment that results in oxidation of ED compounds.

Furthermore, we investigated tyrosinase activity of ED and plasma treated ED in cell-free environment.

### Tyrosinase activity and kinetic studies of ED and PAED

To determine the action of ED and PAED, we checked the mushroom tyrosinase inhibitory activity in a cell-free environment. The tyrosinase inhibitory activity (IC_50_) (IC_50_ is the minimum concentration of a compound required for 50% inhibition of the activity) for as-synthesized 4a, 4b, 4c, 4d, 4e, 6a and 6b ED compounds was found as 25 μg/ml, 29 μg/ml, 15 μg/ml, 58 μg/ml, 19 μg/ml, 5 μg/ml, and 7 μg/ml, respectively (Table 1). Moreover, after plasma treatment for different time intervals, we observed that inhibition activity of 4a, 4b, 4d and 6a was improved more as compared to without treatment. However, compound 6a showed excellent tyrosinase inhibitory activity with the lowest IC_50_, 3 ± 1 μg/ml, among all the synthesized eugenol analogues. On the other hand, highest relative change in IC_50_ value was observed for 4b compound after plasma treatment. The further details in Table 1 show that the inhibition activity of all ED increases after plasma treatment, except for 4c, 4e, and 6b compounds. This shows that the APPJ can influence the tyrosinase activity (either increased or decreased) depending upon the structural changes occurred in ED compounds after treatment. However, we observed the highest relative improvement in IC_50_ value of compound 4b among all ED compounds at 10 min plasma treatment. However, 6a showed excellent tyrosinase inhibitory activity with the lowest IC_50_ value among all the synthesized eugenol analogues before and after plasma treatment. Therefore, we used the 4b and 6a compounds for further investigations, based on the decrease in IC_50_ value to a global minimum of the experiment after plasma treatment.

**Figure 4:**
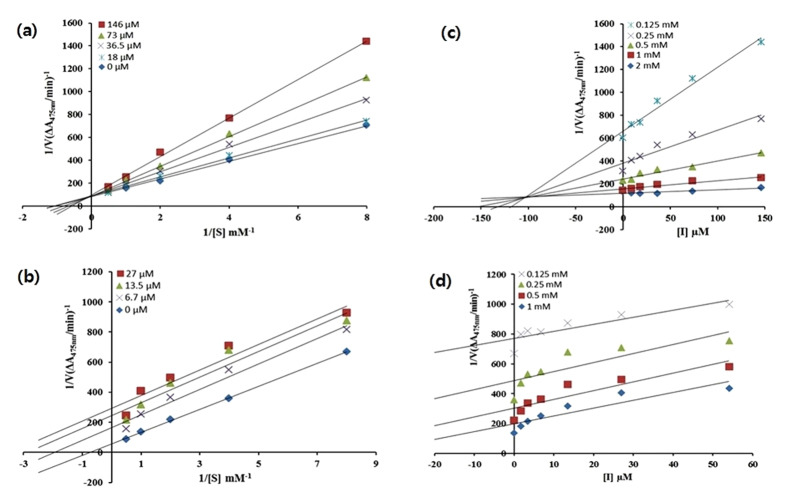
Lineweaver-Burk plots corresponding to various concentrations of compound (**a**) 4b and (**b**) 6a, and Dixon plots corresponding to various concentrations of compound (**c**) 4b and (**d**) 6a, respectively for the inhibition of the diphenolase activity of mushroom tyrosinase in the presence of different concentrations of L-DOPA (0.0, 0.125, 0.25, 0.5, 1.0 and 2.0 mM).

Latter, we studied the kinetic behavior of mushroom tyrosinase during the oxidation of L-DOPA with the selective ED compounds (4b and 6a). In the present investigation, the oxidation reaction of L-DOPA by mushroom tyrosinase follows the Michaelis-Menten kinetics. The inhibitory mechanism of both of the compounds was determined by using Lineweaver-Burk plot (double reciprocal plot) as well as a Dixon plot at various concentrations of substrate L-DOPA (0.0, 0.125, 0.25, 0.5, 1.0 and 2.0 mM), as shown in Fig. 4. The double-reciprocal plot of enzyme activity in the presence of compound 4b with concentration 0.0, 18.0, 36.5, 73, 146 μM and compound 6a with concentration 0.0, 6.7, 13.5, 27 μM are shown in Fig. 4(a,b), respectively. The data are displayed as a plot of inverse velocity (1/V) versus inverse substrate (1/[s]) concentrations. The results show that as the concentrations of compound 4b increases, the Michaelis–Menten constant (K_m_) value of the compound 4b also increases gradually, with a common intercept on the 1/V axis but with different slopes indicating that 4b is a competitive inhibitor of mushroom tyrosinase (Fig. 4(a)). However, an increase in 6a concentrations results in a decrease of V_max_ and K_m_ values (Fig. 4(b,d)), which confirms that compound 6a is an uncompetitive inhibitor. Similar method was used by the previous research groups to show the competitive inhibitor or uncompetitive inhibitor^30^,^31^. The inhibition constant K_i_ (100 μM) of compound 4b was calculated directly from a Dixon plot as shown in Fig. 4(c). The Dixon plot was determined by 1/V versus inhibitor concentrations [I] at various concentrations of substrate in mM. We have used the Kojic acid and tropolone in our present study because they are the most effective tyrosinase inhibitors, due to the presence of copper in tyrosinase enzyme^32^. Further, to determine the action of these eugenol derivatives on the structure of tyrosinase enzyme, we performed CD analysis and molecular docking studies.

### Circular dichroism analysis and molecular docking of ED with tyrosinase enzyme

**Figure 5:**
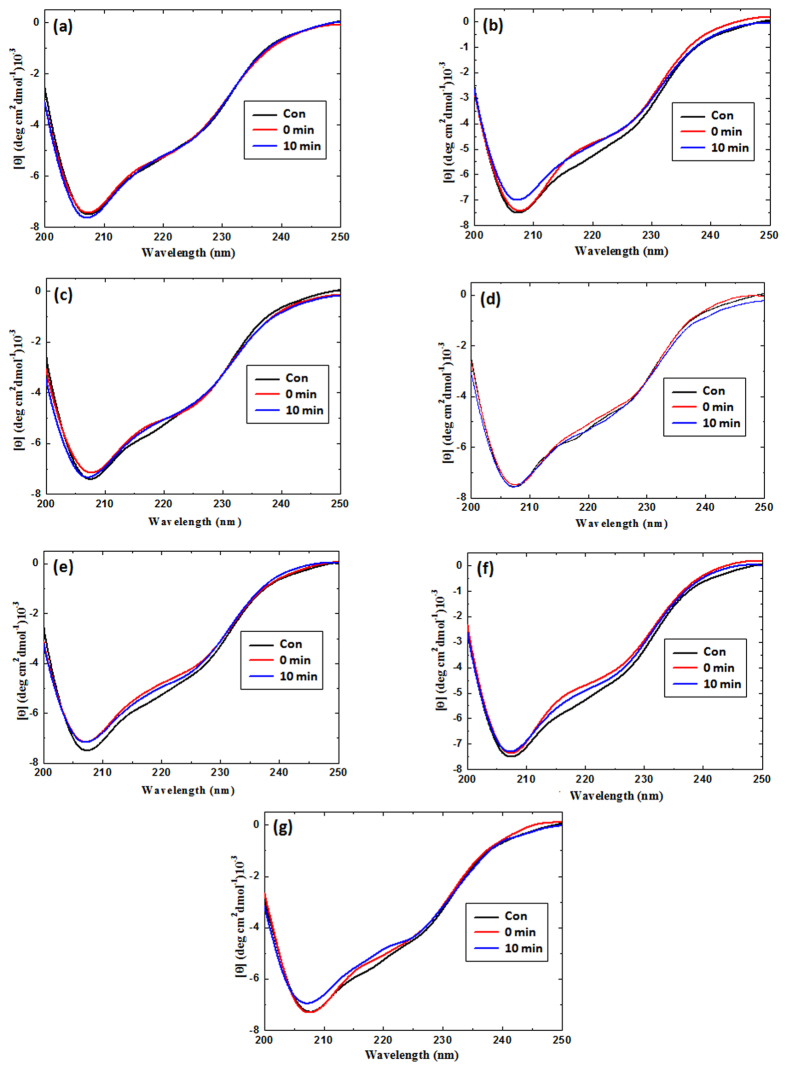
Circular dichroism spectra of ED compounds and plasma treated eugenol derivatives with mushroom tyrosinase. The spectra (**a**) 4a, (**b**) 4b, (**c**) 4c, (**d**) 4d, (**e**) 4e, (**f**) 6a, and (**g**) 6b are shown, respectively. The control spectrum represents the mushroom tyrosinase (black line), tyrosinase with as-synthesized test compounds (red line), and tyrosinase with 10 min plasma-treated test compound (blue line).

The CD spectrum showed slight changes in α-helix and β-sheets in mushroom tyrosinase, after interacting with ED and PAED. The structural changes in tyrosinase after incubation with ED and PAED were plotted in Fig. 5. Figure 5, shows the variations in tyrosinase structure after interacting with ED and PAED and the detailed variations are displayed in Table 2. We observed that the control tyrosinase enzyme has 16% α-helix, 33% β-sheets, 7% turn, and 44% random coil. After the interaction with 4a, the amount of α-helix changed to 15% and β-sheets changed to 35%. On the other hand, after the plasma treatment of 4a the amount of α-helix changed to 17% and β-sheets changed to 31%. While, after interacting with 4b, the amount of α-helix is 16.0%, β-sheets 29%, turn 10%, and random coil is 45.0%. However, plasma-activated 4b interacts differently with tyrosinase enzyme, showing 16% α-helix, 33% β-sheets, 6% turn, and 45% random coil. When 6a ED interacted with tyrosinase enzyme, we observed 20% α-helix, 25% β-sheets, 11.0% turn, and 44% random coil. However, 6a PAED interacts with tyrosinase enzyme, the α-helix changed to 19%, β-sheets 28%, turn 9%, and random coil 44%. This shows that after plasma treatment of ED compounds they interact differently with the tyrosinase enzyme (dependingJ upon the structure changes), therefore no particular trend was observed in CD analysis. To obtain further detail on the interaction of the ED with tyrosinase enzyme, we studied the molecular docking.

**Figure 6:**
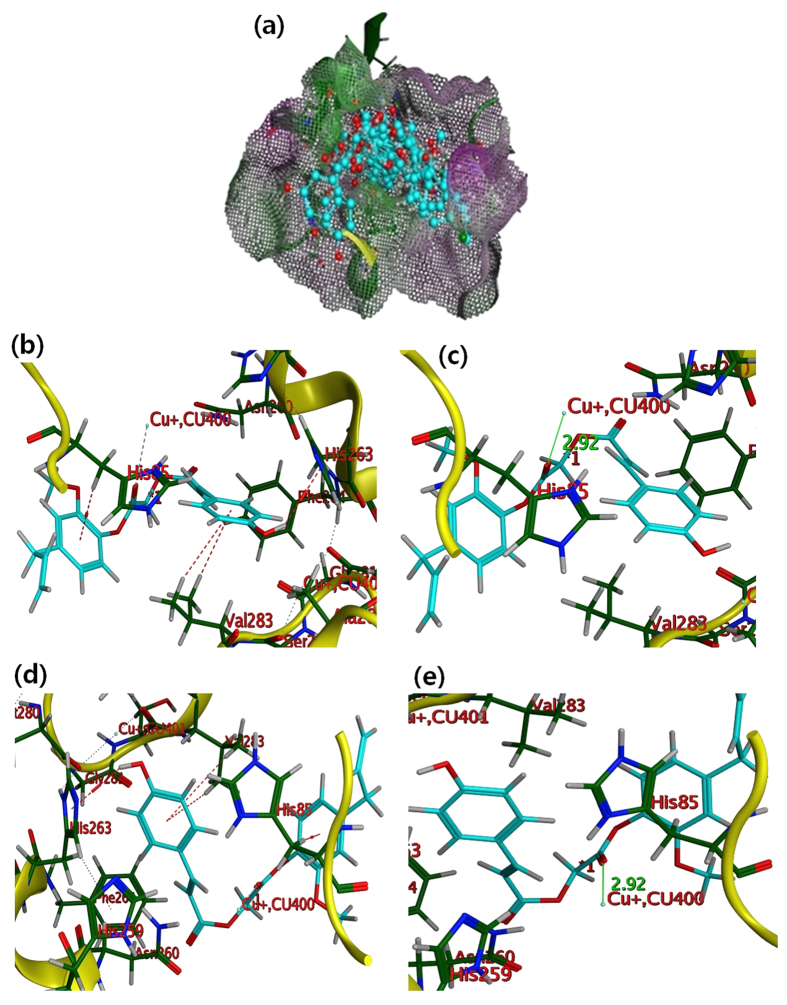
Docking poses of as-synthesized ED compounds in 3D space. (**a**) Overlaying of best docked poses inside the pocket. Ligands and key residues are shown by stick mode, in cyan and green color respectively. Oxygen atoms are shown in red color. (**b–e**) Docking poses of 4b and 6a in 3D space, molecules are shown in cyan color (stick mode) and receptor is shown in yellow ribbons and cartoons. π-H interactions are represented by red dashed lines. Interaction of oxygen atom of ligand with Cu ion is shown in green colored solid line. (**b**) π-H interactions of 4b with His85, Val283 and His263 (**c**) Interaction of ligand 4b with Cu ion, shown in green colored solid line. (**d**) π-H interactions of 6a with His85 and His263 and Val283. (**e**) Interaction of oxygen atom of ligand 6a with Cu ion, shown in green colored solid line.

In order to explain and provide an understanding of the observed tyrosinase inhibitory activity, molecular docking analysis of ED compounds and most active PAED (4b and 6a) was performed. All the docked conformations for each compound were analyzed and it was found that the most favorable docking poses with maximum number of interactions were those which were ranked the highest based on the least energy, which was calculated as a negative value by the software. The most favorable docking poses of the 30 docked conformations for each ED compounds were retained to investigate the interactions of the docked conformations within the active site. All the top ranked docking conformations of ED compounds were stacked well inside the active site cavity. His244, His85, His263, Val283, His296, Asn260, Val248, His260, His261, and Phe264 are found to be key residues, which are also reported in the literature^33^,^34^,^35^,^36^. The active site consisted of both the hydrophobic and hydrophilic amino acids along with the two copper ions. The hydrophobic portion was constructed of Val 283, Met280, Ala246, Phe90, Ala286, Val 248, Phe246, and His256 while the hydrophilic portion contained amino acids including His244, His279, Gly86, His61, Ser282, His 85, His 263, Gly281, Glu256, Asn260, His256, Glu322, Gly96, and Ser282. The active site was a deep bowl shaped cavity with a wide opening. The best positions of the best possible docked poses inside the active site are shown in Fig. 6(a).

In ED docking model, the carbonyl oxygen in ligands 4b and 6a made close contact with the Cu ion (CU:400). Two copper (CU9:400 and CU10:401) ions are part of the active site. CU:400 are shown as cyan dot in Fig. 6(b,c), while both the CU:400 and CU:401 are present in Fig. 6(d,e). However, both the copper ions are shown as grey colored circle in all the images that depict interaction of ligands in 2D space (Figure S3). The docking model represents that the compounds 4b and 6a anchored themselves in active site through π-H interaction with His85, Val283 and His263, represented in red dashed lines as shown in Fig. 6(b,d). Phenyl ring of ligand 4b and 6a established two π-H interactions with side chain H of the Val 283. The reason of the good activities of these both ligands could be attributed to their strong binding with amino acid residues through π-H interaction. Moreover, both the ligands seemed to establish a strong interactions with Cu ion, shown in green colored solid line (Fig. 6(c,e)).

The hydroxyl group of as-synthesized ED compounds made close contacts with key amino acids such as Glu322, His263 and Arg268 (Fig. 6). Binding mode analysis of the best possible binding mode of the most preferred docked conformations revealed that ligands were anchored in the active site through hydrogen bonding, hydrophobic, polar, π-π, and π-H interactions at different potentials. Figure S3(a–g) shows the 2D interactions of all the most favorable docking poses of ED compounds with the enzyme, these interactions are recorded in Table S1(a–d). The aromatic rings made a similar stack of interactions with His263, and Val283 for ED compounds 4a, 4b, 4c and 4d (Figure S3(a–d)). Docking results revealed that the ED might be potent inhibitors of tyrosinase as they showed strong interactions with key residues, which demonstrated their inhibitory ability. In addition, ED compounds showed a good binding free energy (−6.5807 to −5.0116 Kcal/mol) and binding energy (London dG ranging from −9.55 to −7.83 Kcal/mol) as shown in Table S1(a–d). The interaction of ED compounds with amino acid residues in active site is presented in Figure S3(a–g). Polar amino acids are shown in pink color circle, while acidic are again outlined with red color while amino acids basic in character are outlined with blue color. Lipophilic amino acids were shown in green color. Metal ion was shown in grey color. Proximity contour is marked in dotted line. Interactions are shown in dotted lines. Hydrogen bonding was shown in green color when it is with the side chain of amino acid, and shown in blue color when it is established backbone of receptor. The direction of the arrow shows the donor and acceptor (points toward acceptor). Arene-aren and aren-H interactions are also marked with phenyl ring and H. The receptor exposure is given as blue shadow with the circle. All the detailed interactions are recorded in Table S1 of supporting information. It is evident from the theoretical results that synthesized compounds showed comparable interactions and binding energy with those of the Kojic acid (Table S1 and Figure S3). Taking into account the peculiarities of the active site residues in the tyrosinase catalysis, it can be inferred that ED compounds made strong contact with the side chains of tyrosinase. The enhanced affinity of ED towards the residues was reflected by their strong interaction with residues such as His244, His85, His263, Val283, His296, Asn260, Val248, His260, His261, and Phe264. Besides being directly involved in the architecture of the active site, the residue plays a vital role in positioning other key residues in the active site appropriately for the catalysis^33^,^34^,^35^,^36^. These residues established strong interaction with each other through hydrogen binding, van der Waals and other hydrophobic and polar interactions. Residues are aligned around the active site and are available to interact with the ligand due to these interactions.

**Figure 7:**
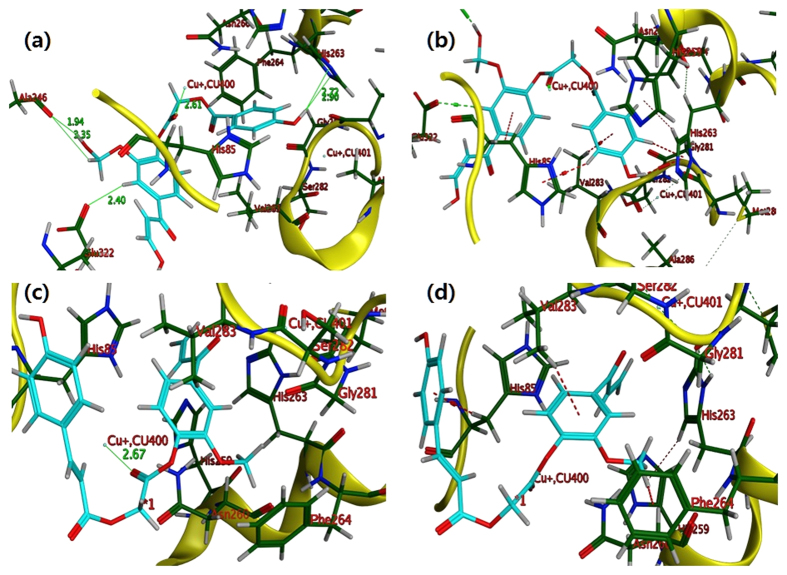
Docking poses of plasma treated ED compounds in 3D space. The molecules are shown in cyan color (stick mode) and receptor is shown in yellow ribbons and cartoons. π-H interactions are represented by red dashed lines. (**a**) Hydrogen bonding and interaction with CU: 400, ligand 4b with His85 and Ala246 shown in green colored solid line. (**b**) π-H interaction of 4b with Val283 and His263 (**c**) Interaction of ligand 6a with CU:400. (**d**) π-H interactions of 6a with His85 and His263 and Val283.

In order to explore further, plasma treated 4b and 6a compounds were subjected to docking simulation with tyrosinase using same parameters as used for ED compounds. In which the active site contained hydrophobic and hydrophilic portion consisting of Val283, Met280, Ala246, Ala 323, Ala246 Val 248, Phe246, His256 and His61, His244, His279, Gly86, Asn81, Ser282, His 85, His 263, Gly281, Glu256, Asn260, His256, Glu322,Glu256, respectively. His244, His85, His263, Val283, His296, Ala323, Glu322, Asn260, Val248, His260, His261, and Phe264 are found to be key residues^33^,^34^,^35^,^36^. In our plasma treated ED docking model, the carbonyl oxygen in ligands 4b and 6a made close contact with the Cu ion (CU:400) as shown in Fig. 7, and Figure S3(i–j). The docking model represents that plasma treated 4b and 6a compounds showed a distinct orientation for interacting with the active site residues of mushroom tyrosinase, both the ligands anchored in active site through π-H interaction. Plasma treated 4b compound anchored itself in active site through four strong π-H interactions with His85, Val283 and His263, represented in red dashed lines as shown in Fig. 7(b). Both the phenyl ring of plasma treated 4b compound established two distant π-H interactions with side chain of the Val 283, His85 while three hydrogen atoms of the phenyl rings also established π-H interactions with the aromatic rings of the His85 and His263. Same ligand anchored itself by establishing five strong hydrogen bonds with active site residues such as His263, Ala323 and Ala246 as shown in Fig. 7(a) and Figure S3(i). Another type of polar interaction between the oxygen atom of the amino acid residues and hydrogen atom of the ligand or vice versa is also noticed in both the plasma treated ligands (4b and 6a) such as ligand plasma treated 4b formulated this interaction with Glu322 and Ala246 as shown in Fig. 7(a) in solid green lines marked with distance. Plasma treated 6a compound anchored itself in the active site through hydrogen bonding, hydrophobic, polar, and π-H interactions at different potentials. The aromatic rings of the ligand 6a made a similar stack of interactions with the side chain of His263, and Val283, for plasma ED compounds however hydrogen atom of the methoxy group present at the phenyl ring also established π-H interactions with phenyl ring of the Phe246 as shown in Fig. 7(d). Figure S3(I,j) showed the 2D interactions of both plasma treated 4b and 6a ED compounds with the key amino acid residues, which are recorded in Table S1(e). Overall docking results revealed that the plasma treated ED might be more potent inhibitors of tyrosinase as they showed more strong interactions (through copious hydrogen bonding, π-H interactions in addition to polar, ionic hydrophobic and hydrophilic interactions) with key residues such as His244, His85, His263, Val283, His296, Ala323, Glu322, Asn260, Val248, His260, His261, and Phe264. Moreover, these interactions are noticed to be intense in addition to the greater in number for plasma treated ED compounds (4b and 6a) as compared to without plasma treated ED compounds (4b and 6a).

Further, we checked the action of ED compounds on murine melanoma (B16F10) cells to determine the melanogenesis.

### Cell viability and melanin detection using ED and PAED

**Figure 8:**
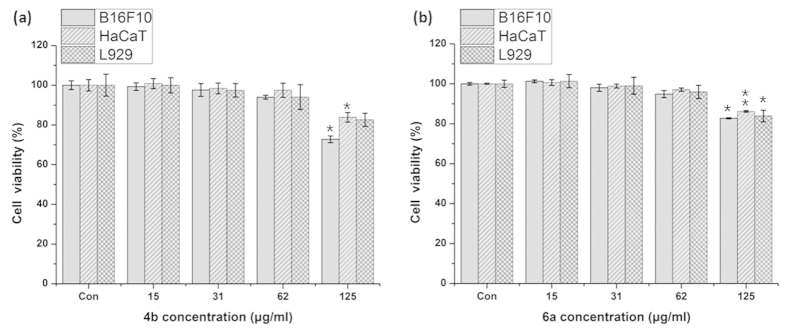
Cell viability measurement. The murine melanoma (B16F10), human skin keratinocyte (HaCaT), and murine skin fibroblast (L929) cells were incubated with different concentrations of (**a**) 4b and (**b**) 6a, and the cell viability was accessed by MTT assay. The data were expressed as a percentage of the control (normalized to 100%) from three independent experiments with mean ± standard deviation and were analyzed using Student’s t-tests, *p < 0.05, **p < 0.005.

To investigate the effect of ED on melanogenesis, we first evaluated the safe dose of test compounds using MTT cell viability assay. For this viability assay, murine melanoma (B16F10) cells with two healthy cell lines, murine skin fibroblast (L929), and human keratinocyte (HaCaT) were incubated with various concentrations of test compounds for 24 hr. MTT assay was then conducted as described in the methods section. The result showed concentration dependent effect of ED compounds as shown in Fig. 8 and Figure S4. However, the viability for all cells types was above 95% at 45 μg/ml concentrations of tested compounds. Therefore, we selected a safer concentration (maximum 42 μg/ml) for further analysis. Figure S5 shows no effect on cell morphology of B16F10 cells after incubation with ED (4b and 6a).

**Figure 9:**
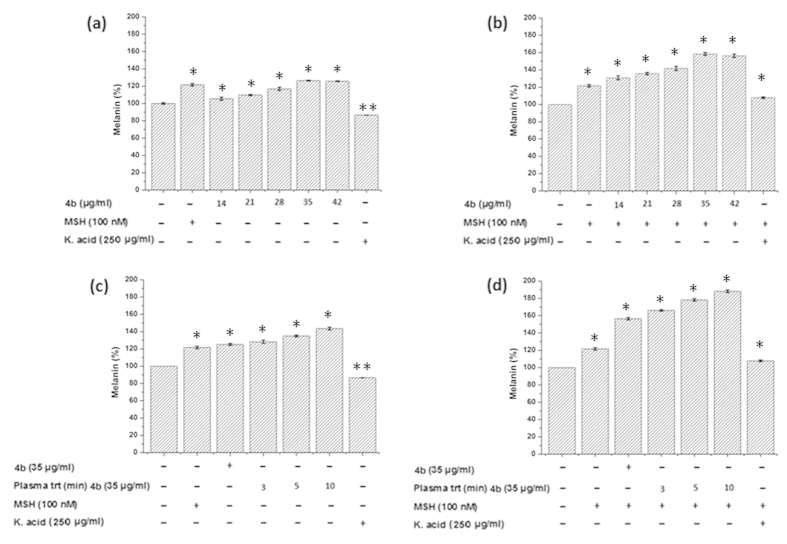
Intracellular melanin measurement. B16F10 cells were incubated with various concentrations of as-synthesized 4b in the (**a**) absence and (**b**) presence of α-MSH and melanin was detected. Moreover, B16F10 cells were also incubated with plasma-treated 4b in the (**c**) absence and (**d**) presence of α-MSH, except for the control containing only the media. All data were expressed as a percentage of the control (normalized to 100%) from three independent experiments with mean ± standard deviation and were analyzed using Student’s t-tests, *p < 0.05, **p < 0.005.

**Figure 10:**
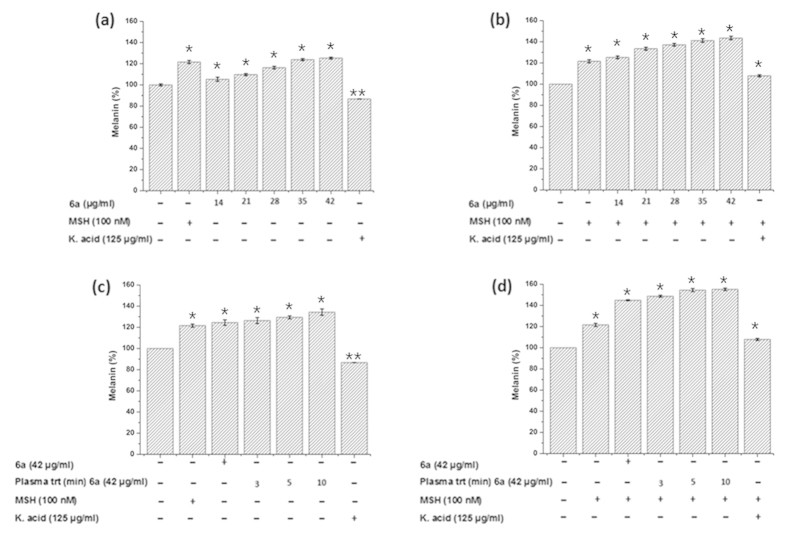
Intracellular melanin measurement. B16F10 cells were incubated with various concentrations of as-synthesized 6a in the (**a**) absence and (**b**) presence of α-MSH and melanin was detected. Moreover, B16F10 cells were also incubated with plasma-treated 6a in the (**c**) absence and (**d**) presence of α-MSH, except for the control containing only the media. All data were expressed as a percentage of the control (normalized to 100%) from three independent experiments with mean ± standard deviation and were analyzed using Student’s t-tests, *p < 0.05, **p < 0.005.

For the melanin study, B16F10 cells were incubated in media containing various concentrations of ED. Each of the ED incubation with cells showed increase in the melanin synthesis (Figs 9,10 and Figure S6). Melanin increase was 116%, 117%, 110%, 115%, 119% of control (normalized to 100%) for ED 4a, 4c, 4d, 4e, and 6b, respectively (Figure S6). However, the highest melanin synthesis was observed for 4b (126%) and 6a (125%) as shown in Figs 9 and 10, respectively. Therefore, we selected 4b and 6a compounds for further detailed melanin study. The test compounds 4b and 6a showed a synergic effect for melanin synthesis when treated with plasma. Further, we tested the action of ED (4b and 6a) and PAED (4b and 6a) in B16F10 cells with alpha-melanocyte-stimulating hormones (α-MSH). α-MSH is well known to activate the melanin synthesis in cells. Hence, using the combined action of ED (4b or 6a) + α-MSH and PAED (4b or 6a) + α-MSH, we can determine if the actions for melanin synthesis are synergetic or antagonistic. If the melanin content without the test compound in the control is taken to be 100%, it was increased to 125% and to 156% by the addition of 4b (35 μg/ml) in the absence or presence of α-MSH, respectively (Fig. 9(a,b)). Interestingly, plasma treatment to 4b (35 μg/ml) also activated its dose-dependent melanin stimulating ability in B16F10 cells (Fig. 9(c,d)). The melanin stimulatory ability of 4b (35 μg/ml) alone was 125% with respect to the control sample which improved to 143% after plasma treatment for 10 min, as shown in Fig. 9(c). A dramatic increase in melanin synthesis up to 188% was observed when melanoma cells were incubated with 10 min plasma-treated 4b compound in the presence of α-MSH (Fig. 9(d)). This confirms the synergic effect of plasma on test compounds with α-MSH. Melanin stimulation was observed when B16F10 cells were incubated with the 6b compound with or without plasma treatment in the presence or absence of α-MSH, as shown in Fig. 10. We used Kojic acid as the negative control. Kojic acid (250 μg/ml) inhibited the melanin synthesis by around 12% compared to the control in the absence of α-MSH. To understand the mechanism of 4b and 6a compounds before and after plasma treatment, we studied the intracellular ROS, cAMP, microphthalmia-associated transcription factor (MITF), tyrosinase and tyrosinase-related protein-1 (TRP1).

### Intracellular ROS, cAMP, MITF, Tyrosinase and TRP1 analysis in the presence of eugenol derivatives (4b and 6a) with or without plasma treatment

**Figure 11:**
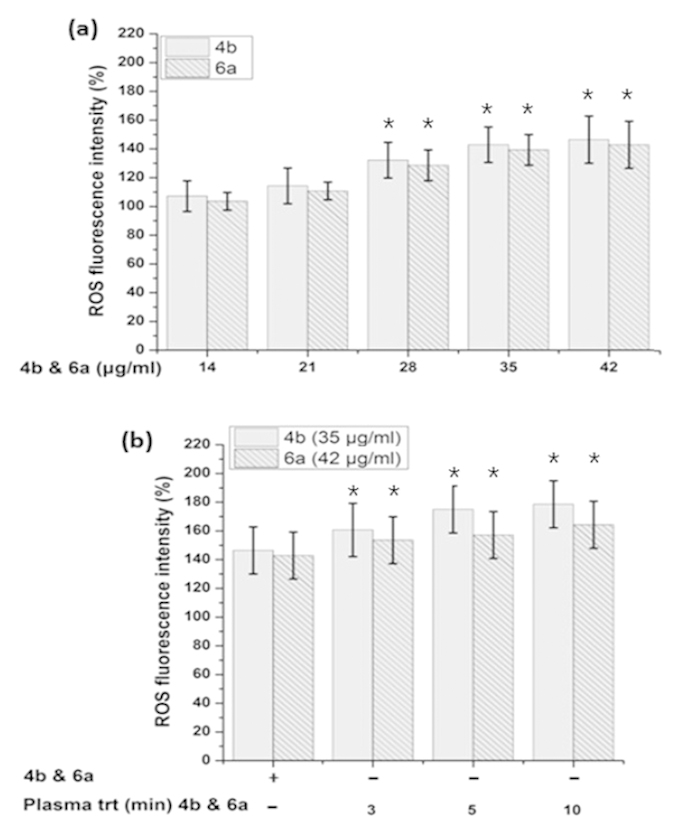
Intracellular ROS detection. The ROS detected from B16F10 cells, incubated with (**a**) as-synthesized and (**b**) plasma-treated 4b and 6a compounds. The data were expressed as a percentage of the control (normalized to 100%) from three independent experiments with mean ± standard deviation and were analyzed using Student’s t-tests, *p < 0.05.

To understand the mechanism for melanin synthesis inside the cells, we selected the most active ED (4b and 6a) for further investigation. Intracellular ROS generation after ED incubation with cells was checked by using H_2_DCFDA. We found increasing intracellular ROS levels with the addition of increasing concentrations of ED compounds (Fig. 11). Further, we compared the intracellular ROS levels for ED and PAED compounds and we found higher intracellular ROS levels for PAED (4b and 6a) as compared to ED compounds (4b and 6a) (Fig. 11(a,b)). Moreover, the 4b compound has shown higher intracellular ROS than 6a, independent of plasma treatment.

**Figure 12:**
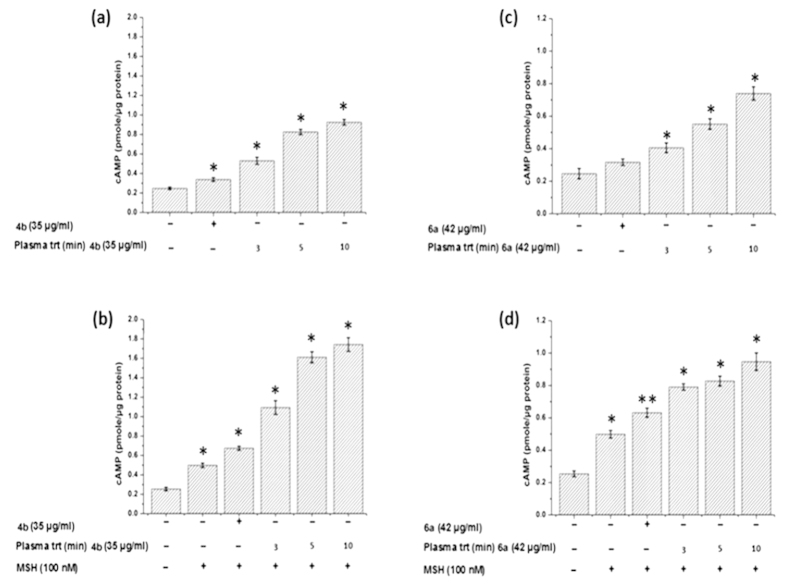
Analysis of cAMP expression in B16F10 cells incubated with (**a**) 4b with and without plasma-treatment, (**b**) 4b with and without plasma-treatment + α-MSH, (**c**) 6b with and without plasma-treatment, and (**d**) 6b with and without plasma-treatment + α-MSH as indicated in graph. The data from three independent experiments was expressed as mean ± standard deviation and was analyzed using Student’s t-tests, *p < 0.05, **p < 0.005.

It is generally known that ROS participates in various cell signaling pathways including cAMP which is important for melanin synthesis^3^,^4^,^8^,^9^. Therefore, we quantified intracellular cAMP in B16F10 cells incubated with 4b and 6a before and after plasma treatment in the presence or absence of α-MSH. The cAMP level was increased with 4b and 6a incubation with cells, compared to the control samples (having media only with cells) as shown in Fig. 12. The cAMP level was enhanced even further when the B16F10 cells were incubated with PAED (4b or 6a), and this enhancement was significantly higher than the control (Fig. 12(a,c)). However, the cAMP level was higher in the presence of α-MSH even at the same dose of plasma treatment to each test compound, as shown in Fig. 12(a,b) for compound 4b and Fig. 12(c,d) for compound 6a. This confirms the synergic effect of plasma and α-MSH with ED for cAMP level that is similar to their synergic effect observed for melanin synthesis in B16F10 cells (Figs 9 and 10). In general, the level of cAMP was higher for 4b than 6a at tested concentrations in the presence or absence of α-MSH.

**Figure 13:**
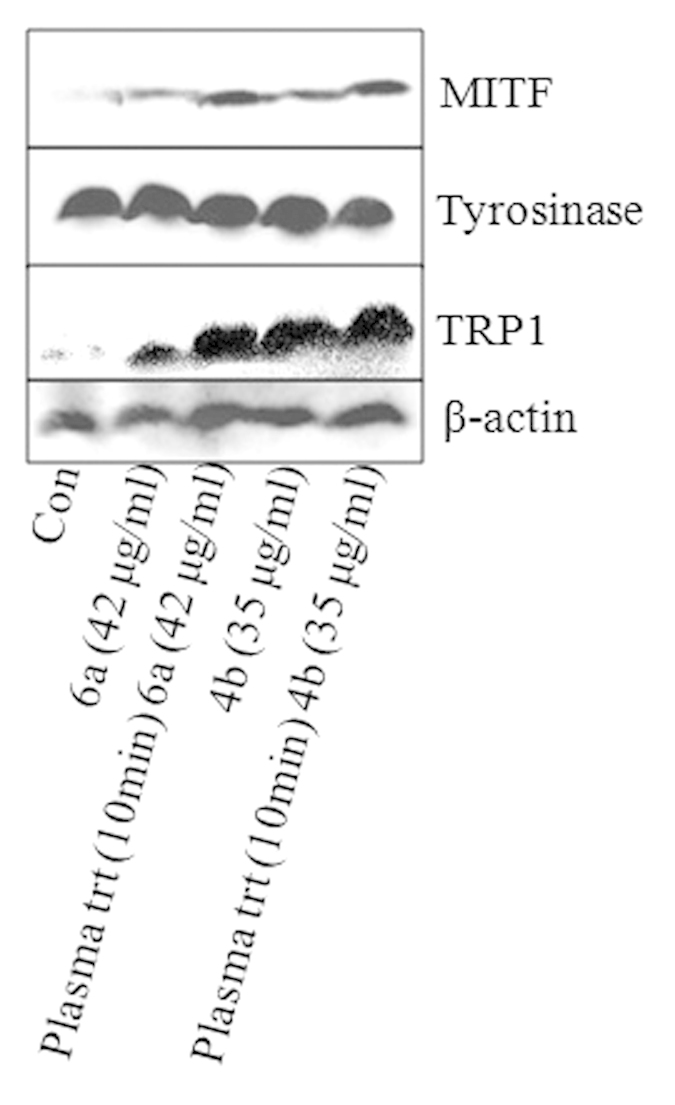
Analysis of MITF, tyrosinase and TRP1 expression in B16F10 cells. The cells were incubated with as-synthesized and plasma treated 4b and 6a compounds, and the expression of indicated proteins was checked by western blot technique.

To elucidate the mechanism of the influence of ED (4b and 6a) and PAED (4b and 6a) on melanogenesis, we have studied the expression of MITF, tyrosinase, and TRP1 in B16F10 cells by western blotting. The cells incubated with ED and PAED (4b or 6a) showed higher expression of MITF and TRP1 than the control. However, the cells incubated with PAED showed more expression of MITF and TRP1 than for ED for both 4b and 6a compounds (Fig. 13). However, no change was observed in cellular tyrosinase expression after incubation with ED and PAED from that of the control, as illustrated in Fig. 13.

## Discussion

In present work, we synthesized eugenol derivatives (ED) with the aim of inhibiting the tyrosinase activity, which we predicted might result in decrease of melanin synthesis inside the cells. However, our investigations found that the ED compounds inhibit tyrosinase activity in cell-free system (Table 1) while activate melanin synthesis in cell culture system. Furthermore, we treated these compounds with N_2_ atmospheric pressure plasma jet for 3, 5, and 10 min, and checked the activity again. Plasma treatment improved the tyrosinase inhibition activity (IC_50_) of 4a, 4b, 4d, and 6a compounds. Interestingly, we observed the highest relative improvement in IC_50_ value of compound 4b among all eugenol derivatives at 10 min plasma treatment. However, 6a showed excellent tyrosinase inhibitory activity with the lowest IC_50_ value among all the synthesized eugenol analogues before and after plasma treatment. Therefore, we selected 4b and 6a compounds for further investigations, based on the decrease in IC_50_ value to a global minimum of the experiment after plasma treatment. For detailed investigation, we performed kinetic study of selected compounds (4b and 6a). Our result showed that the tyrosinase inhibition efficiency of 4b and 6a increases after the plasma treatment. In other words, we can say that the PAED (4b and 6a) are more efficient tyrosinase inhibitors than their ED counterpart in a cell-free environment. Similar results were also observed by Kim *et al.*^29^, where plasma treatment increased the tyrosinase activity of a natural compound naringin.

For further investigation, we performed CD experiments and observed that these ED compounds have the ability to interact with the secondary structure of proteins. Also, the interactions were observed to be different before and after plasma treatment to ED. These interactions do not show major changes in the secondary structure of tyrosinase enzyme. These results were also supported by Ghebi *et al.*^37^, where they observed that n-alkyl sulfates cause only minor conformational changes in the tyrosinase enzyme in CD analysis, but they observed changes in the enzyme’s function. Later, we performed molecular docking to determine the binding sites of tyrosinase with these ED compounds. The binding mode inspection and evaluation of the docking poses revealed the fact that the tested compounds (4b and 6a) happened to manage the deterministic and greater number of interactions including strong hydrogen bonding, π-cation interactions, hydrophobic and polar interactions after plasma treatment as compared to their synthesized counterpart (Figs 6 and 7, Figure S3, and Table S1). Both the plasma treated ligands turn out to establish copious π-cation interactions in addition to polar and hydrophobic attractions as shown in Fig. 7(b,d) and Figure S3. Plasma treated compound 4b happened to anchor itself tightly inside the active site gorge through multiple strong hydrogen bonds (Fig. 7(a) and Figure S3) and it showed good binding score (−6.8072 Kcal/mol) and binding energy (London dG −9.9701 Kcal/mol), as compare to their counterpart ED compound (−6.4590 Kcal/mol and −8.7758 Kcal/mol) (Table S1). Both, the interactions and binding energies of the ligand 4b have been improved after plasma treatment (Fig. 7(a,b), Figure S3, Table S1), which proclaim that plasma treated 4b compound is better inhibitor than without plasma treated 4b. Similarly plasma treated compound 6a, showed higher binding score (−6.8403 Kcal/mol in S field) and binding energy (London dG −9.1881 Kcal/mol) than its synthesized counterpart (which shows binding score as −6.5807 Kcal/mol, and binding energy as London dG **−**8.7758 Kcal/mol). Orientation of the docking pose of the plasma treated ligand 6a inside the active site was found to be quite similar to that of the plasma treated ligand 4b and both the ligands appeared to follow the true key and lock mechanism of the docking phenomena, which resulted in tremendous interactions^38^. The plasma treated ligand 6a successfully established the remarkably strong hydrogen bonding of 2.89 Å, 3.63 Å, 2.97 Å and 3.81 Å with the Ala246, His263, Ala323 and Glu322 respectively at multiple ends of the ligand which bind it tightly inside the active site (Fig. 7(c,d) and Figure S3). Hydrogen bonding and other ligand protein interactions are thought to be strong determinant for the inhibition properties of the compounds^38^,^39^. Docking computations of the plasma treated compounds revealed the fact that the binding scores, binding energies and interactions with amino acids improved which could impart better inhibition potential to the ligands under discussion. The reason for the strong and greater number of interactions of the tested molecules after plasma treatment especially 4b, can be attributed to the addition of functional group and increase in the size of the molecule, which are indicators for better inhibition activity due to the availability of the greater number of the interaction sites^39^.

Furthermore, we investigated the reason for the enhanced activity of ED compounds after plasma treatment. Various research groups^40^,^41^,^42^ have shown that the input of energy can induce structural changes in natural compounds and molecules. Stereospecificity in hydroxyl radical scavenging activities of four ginsenosides was reported by heat processing^40^,^41^. It was found that a double bond at carbon-20 (22), or the OH group at carbon-20 that is geometrically close to the OH at carbon-12, increased the OH scavenging activity of ginsenosides upon heat processing^40^. Radiolytic transformation of rotenone with potential anti-adipogenic activity has also been reported^41^. Similarly, the enhancement of pancreatic lipase inhibitory activity of curcumin by radiolytic transformation has been reported^42^. Furthermore, irradiations can produce the radiolytic transformation of rotenone and curcumin to produce new compounds^41^. Therefore, the plasma being the source of energy, free radicals, ions, and molecules^43^ may also induce modification in the ED structure that may increase its efficiency. For better understanding the chemical change in tested ED compounds (4b and 6a) after plasma treatment we used NMR spectroscopy. In case of compound 4b the groups which may propose after plasma treatment are more susceptible for oxidation as compared to the functional group present in compound 6a. Tyrosinase enzyme may preferably bind with the compounds that are easily oxidized (the substrates of tyrosinase enzymes are tyrosine and L-DOPA both possess phenolic –OH groups that are easily to be oxidized). While, in some ED compounds the structure destroy or modified lot after the plasma treatment in those compounds we observed the loss in activity. Whereas, in the case the structure has not modified/distroyed to large extent after plasma exposure, only some change in functional group was observed in those cases the activity was improved as we see for 4b and 6a ED compounds. The structural changes observed in NMR of the selected compounds (4b and 6a) are shown in Figure S2 and proposed structures are given in Fig. 3.

As a first step toward finding possible biomedical applications, we further tested the cytotoxicity of all ED compounds using skin melanoma (B16F10), skin fibroblast (L929) and human skin epidermal keratinocyte (HaCaT) cells. We found that all ED compounds are safe up to 42 μg/ml concentration with no cytotoxicity. Therefore, we used this concentration (42 μg/ml) as a maximum concentration for the melanin test in B16F10 cells for all as-synthesized ED compounds. We observed that the melanin content was increased in the cells with increasing concentrations of ED, while the highest melanin content was observed for 4b and 6a compounds. The visual test results shown in Figure S7 confirm the intracellular melanin improvement after 4b and 6a incubation with cells. We treated 4b and 6a ED compounds with plasma, and tested them for melanin synthesis in B16F10 cells as shown in Figs 9 and 10. These results show that melanin synthesis was enhanced even more by plasma treatment to the test compounds.

Further, to understand the mode of action of ED and PAED on melanin synthesis inside the cells we have tested the intracellular ROS, cAMP, MITF, TRP1 and Tyrosinase expression. According to previously reported work, the decrease in ROS/RNS formation by the promotion of antioxidant systems such as GSH or GPx is associated with the reduction of melanogenesis in cultured melanoma cells and human melanocytes by inhibiting tyrosinase activity^44^,^45^. Similarly, Chou *et al.*^46^ and Yanase *et al.*^47^ reported that ROS was directly involved in cell signaling for melanin synthesis. These studies found that the decrease in ROS levels is correlated with down-regulation of melanogenic signaling pathways such as cAMP and MITF that subsequently reduce the tyrosinase activity and TRP1 expression finally contributing in melanin inhibition. Conversely, the increases in ROS level can up-regulate the melanogenic signaling pathways and enhance the melanin production^48^. Our present results confirm that the ED (4a and 6b) increased the intracellular ROS with increasing concentration (Fig. 11). The increased ROS level activated the cAMP pathways resulting in the stimulation of MITF and TRP1 expressions (Figs 10 and 13), that eventually up-regulate melanin synthesis. Briefly, we found increase in the expression of cAMP, MITF, and TRP1 but not tyrosinase expression after cellular incubation with ED (4a and 6b) and PAED (4a and 6b). A recent report^49^ represents similar results in which Melia azedarach (MA) extract increased the melanin content in B16F10 cells without affecting intracellular tyrosinase. Conversely, the Melia azedarach (MA) extract remarkably increased the expression of TRP1 protein. Therefore, they concluded that TRP1 is responsible for higher melanin production^49^. Interestingly, in another study the down-regulation of TRP1 was reported to be involved in melanin inhibition but there was no significant change in the expression of tyrosinase^50^. This represents the pivotal role of TRP1 in melanogenesis and TRP1 may increase the melanin synthesis without tyrosinase contribution. We observed similar behavior of 4b and 6a before and after plasma treatment, where we did not observe the change in the tyrosinase expression however, TRP1 was expressed remarkably. Thus, melanin production is stimulated by up-regulation of TRP1 in our study. Indeed, we demonstrated that the up-regulation of TRP1 along with cAMP, and MITF by tested ED and PAED, enhanced the melanin synthesis. Interestingly, the cAMP, MITF and TRP1 expressions were higher for PAED (4b and 6a) than ED (4b and 6a). This could be the reason for more melanin content in the cells incubated with the tested PAED (4b and 6a) than ED (4b and 6a).

Our results confirmed the previous findings in which opposite melanogenic responses of compounds were observed in cell free environment and in cell culture environment^51^,^52^. It is recently reported that fucoidan compounds decreased the tyrosinase activity in cells, but did not directly alter the mushroom tyrosinase activity in a cell-free system^53^. Similar results were observed in another report in which increasing concentration of docosahexaenoic acid (DHA) reduced tyrosinase activity within the cells, but DHA failed to reduce mushroom tyrosinase activity in a cell-free environment^54^. Additionally, a chemical compound arbiutin is reported with opposite behavior in cell free system and cell culture system^51^,^52^. This discrepancy in the results is because of different conditions of cell free system and cell culture system^51^. Maeda and Fukuda^51^ also claim that the inhibition of crude mushroom tyrosinase activity (cell free environment) may not correlate with the inhibition of tyrosinase or melanin production in cultured cells, and to evaluate the effectiveness of a compound, it is necessary to access the cellular activity and not to relay only on the activity obtained in cell free environment^51^.

Hence, our present work demonstrates that plasma can modify the structure of the synthesized 4b and 6a ED compounds in order to increase their efficiency. This study provides new plasma chemistry that can help to increase the utility of plasma medicine.

## Materials and Methods

### Experimental

All chemicals used for the synthesis of compounds, mushroom tyrosinase and L-DOPA were purchased from Sigma Chemical Co. USA. Melting points were determined using a Digimelt MPA 160, USA melting point apparatus, and are reported uncorrected. The FTIR spectra were recorded with a Shimadzu FTIR–8400S spectrometer (Kyoto, Japan, υ, cm^−1^). The ^1^H NMR and ^13^C NMR spectra (CDCl_3_) and (DMSO-*d*_6_) were recorded using a Bruker 400 MHz spectrometer. Chemical shifts (δ) are reported in ppm downfield from the internal standard tetramethylsilane (TMS). The purity of the compounds was checked by thin layer chromatography (TLC) on a silica gel plate using n-hexane and ethyl acetate as the mobile phase. The procedure for the synthesis of the desired compounds was depicted in Fig. 1.

### Synthesis of eugenol chloroacetyl derivative (2).

A mixture of eugenol (1) (0.01 mol) and triethylamine (0.01 mol) in anhydrous dichloromethane (25 ml) was cooled in an ice salt mixture to 0 to −5 °C. Chloroacetyl chloride (0.01 mol) in dry dichloromethane was added drop wise to this reaction mixture with constant stirring over a period of 1 hr while maintaining a constant temperature. The reaction mixture was then stirred at room temperature for a further 5 hr, and then washed with 5% HCl and 5% sodium hydroxide solution. The organic layer was washed with saturated aqueous NaCl, dried over anhydrous magnesium sulphate, and filtered. The solvent was then removed under reduced pressure. The crude product was purified by a silica gel column to afford the corresponding eugenol chloroacetyl derivative (2). Colorless oil; reaction time, 6 hr; yield, 84; R_f_ 0.61 (n-hexane: ethyl acetate 3:1), FTIR ν_max_ cm^−1^: 3025 (sp2 C-H), 2973 (sp3 C-H), 1728 (C = O ester), 1615 (C = C aromatic), 1158 (C-O, ester).

### Synthesis of eugenol analogues (4a-4e) and (6a-6b)

A mixture of eugenol chloroacetyl derivative (2) (0.01 mol), hydroxy substituted benzoic acids (3a-3e) (0.01 mol), triethyl amine (0.01 mol), and potassium iodide (0.01 mol) in dimethyl formamide (25 ml) was stirred overnight at room temperature (Fig. 1(a)). The reaction mixture was poured into finely crushed ice while stirring and extracted with ethyl acetate (4 ×25 ml). The combined organic layer was washed with 5% HCl and 5% sodium hydroxide, and finally with aqueous NaCl solution. The organic layer was dried over anhydrous magnesium sulphate and filtered, and the solvent was then removed under reduced pressure to reveal the crude products (4a-4e). The title compounds (4a-4e) were purified by silica gel column chromatography. The same procedure was used for the preparation of compounds (6a-6b) (Fig. 1(b)).

**2-[2-methoxy-4-(prop-2-en-1-yl)phenoxy]-2-oxoethyl 3-hydroxybenzoate (4a)** solid; reaction time, 24 hr; yield, 76%; melting point, 70–72 °C; R_f_ 0.48 (n-hexane:ethyl acetate 2:1), FTIR ν_max_ cm^−1^: 3124 (O-H), 3965 (sp2 C-H), 2829 (sp3 C-H), 1705 (C = O ester), 1602 (C = C aromatic), 1129 (C-O, ester); ^1^H NMR (CDCl_3_, δ ppm): 7.71 (dd, *J* = 5.2, 1.6 Hz, 1H, H-6), 7.58 (d, *J* = 1.6 Hz, 1H, H-3′), 7.36 (t, *J* = 8.0 Hz, 1H, H-5), 7.09 (dd, *J* = 1.6, 1.2 Hz, 1H, H-2), 7.02 (d, *J* = 8.0 Hz, 1H, H-6′), 6.80 (m, 2H, H-4, H-5′), 5.98 (m, 1H, H-8′), 5.15 (s, 2H, -CH_2_), 5.09 (m, 2H, H-9′), 3.84 (s, 3H, -OCH_3_), 3.40 (d, *J* = 6.8 Hz, 2H, H-7′), 1.62 (s, 1H, -OH); ^13^C NMR (CDCl_3,_ δ ppm); 166.1 (C = O ester), 165.5 (C = O, ester), 155.6 (C-3), 150.6 (C-1′), 139.4 (C-2′), 137.3 (C-5′), 136.9 (C-6), 130.5 (C-2), 129.7 (C-1), 122.5 (C-3′), 122.3 (C-5), 120.7 (C-4′), 120.6 (C-9′), 116.5 (C-6′), 116.2 (C-8′), 112.8 (C-4), 61.0 (-OCH_3_), 55.9 (-CH_2_), 40.0 (C-7′).

**2-[2-methoxy-4-(prop-2-en-1-yl)phenoxy]-2-oxoethyl 4-hydroxybenzoate (4b)** solid; reaction time, 24 hr; yield, 82%; melting point, 81–83 °C; R_f_ 0.50 (n-hexane:ethyl acetate 2:1), FTIR ν_max_ cm^−1^: 3126 (O-H), 2956 (sp2 C-H), 2876 (sp3 C-H), 1705 (C = O ester), 1589 (C = C aromatic), 1148 (C-O, ester); ^1^H NMR (DMSO-*d*_6_, δ ppm): 10.46 (1H, s, -OH), 7.87 (d, *J* = 9.2 Hz, 2H, H-2, H-6), 7.02 (d, *J* = 8.0 Hz, 2H, H-3, H-5), 6.95 (s, 1H, H-3′), 6.86 (d, *J* = 8.4, 1H, H-6′), 6.77 (d, *J* = 8.0 Hz, 1H, H-5′), 6.00 (m, 1H, H-8′), 5.10 (s, 2H, -CH_2_), 5.04 (m, 2H, H-9′), 3.74 (s, 3H, -OCH_3_), 3.14 (d, *J* = 4.8 Hz, 2H, H-7′); ^13^C NMR (DMSO-*d*_6,_ δ ppm); 166.2 (C = O ester), 160.7 (C = O, ester), 151.5 (C-4), 140.2 (C-1′), 137.0 (C-2′), 136.8 (C-5′), 132.4 (C-3, C-5), 132.2 (C-1), 122.4 (C-3′), 120.8 (C-4′), 116.2 (C-6′), 115.4 (C-2,C-6), 112.9 (C-9′), 111.8 (C-8′), 60.8 (-OCH_3_), 56.0 (-CH_2_), 40.1 (C-7′).

**2-[2-methoxy-4-(prop-2-en-1-yl)phenoxy]-2-oxoethyl 2,4-dihydroxybenzoate (4c)** solid; reaction time, 24 hr; yield, 76%; melting point, 99–101 °C; R_f_ 0.46 (n-hexane:ethyl acetate 2:1), FTIR ν_max_ cm^−1^: 3093 (O-H), 2952 (sp2 C-H), 2852 (sp3 C-H), 1710 (C = O ester), 1602 (C = C aromatic), 1159 (C-O, ester); ^1^H NMR (DMSO-*d*_6_, δ ppm): 7.73 (d, *J* = 8.8 Hz, 1H, H-6), 7.04 (d, *J* = 9.6 Hz, 1H, H-6′), 6.98 (d, *J* = 1.6 Hz, 1H, H-3), 6.80 (dd, *J* = 6.4, 1.6Hz, 1H, H-5), 6.43 (dd, *J* = 2.4, 6.4 Hz, 1H, H-5′), 6.34 (d, *J* = 2.4 Hz, 1H, H-3′), 5.99 (m, 1H. H-8′), 5.18 (s, 2H, -CH_2_), 5.06 (m, 2H, H-9′), 3.76 (s, 3H, -OCH_3_), 3.37 (d, J = 6.8 Hz, 2H, H-7′); ^13^C NMR (DMSO-*d*_6,_ δ ppm); 168.3 (C = O ester), 166.6 (C = O ester), 165.1 (C-2), 163.2 (C-4), 150.9 (C-1′), 145.1 (C-2′), 139.8 (C-5′), 137.8 (C-6), 132.4 (C-4′), 130.9 (C-3′), 122.8 (C-6′), 120.9 (C-9′), 116.5 (C-3), 115.7 (C-8′), 113.5 (C-5), 109.0 (C-1), 61.1 (-OCH_3_), 56.2 (-CH_2_), 56.0 (C-7′).

**2-[2-methoxy-4-(prop-2-en-1-yl)phenoxy]-2-oxoethyl 3,4-dihydroxybenzoate (4d)** solid; reaction time, 24 hr; yield, 80%; melting point, 148–151 °C; R_f_ 0.42 (n-hexane:ethyl acetate 2:1), FTIR ν_max_ cm^−1^: 3126 (O-H), 2965 (sp2 C-H), 2827 (sp3 C-H), 1712 (C = O ester), 1604 (C = C aromatic), 1128 (C-O, ester); ^1^H NMR (DMSO-*d*_6_, δ ppm): 7.41 (m, 2H, H-5, H-6), 7.04 (d, *J* = 8.0 Hz, 1H, H-6′), 6.97 (d, *J* = 1.6 Hz, 1H, H-2), 6.85 (dd, *J* = 8.4, 1.6 Hz, 1H, H-5′), 6.78 (d, *J* = 1.6 Hz, 1H, H-3′), 5.99 (m, 1H. H-8′), 5.13 (s, 2H, -CH_2_), 5.05 (m, 2H, H-9′), 3.75 (s, 3H, -OCH_3_), 3.35 (d, *J* = 6.8 Hz, 2H, H-7′); ^13^C NMR (DMSO-*d*_6,_ δ ppm); 166.9 (C = O ester), 165.5 (C = O ester), 151.4 (C-3), 150.9 (C-4), 145.6 (C-1′), 139.7 (C-2′), 137.8 (C-5′), 137.2 (C-1), 122.8 (C-6), 122.7 (C-4′), 120.8 (C-3′), 119.8 (C-6′), 116.9 (C-9′), 116.5 (C-2), 115.9 (C-5), 113.5 (C-8′), 60.9 (-OCH_3_), 56.2 (-CH_2_), 40.6 (C-7′).

**2-[2-methoxy-4-(prop-2-en-1-yl)phenoxy]-2-oxoethyl 3,5-dihydroxybenzoate (4e)** solid; reaction time, 24 hr; yield, 74%; melting point, 107–109 °C; R_f_ 0.46 (n-hexane:ethyl acetate 2:1), FTIR ν_max_ cm^−1^: 3125 (O-H), 2924 (sp2 C-H), 2852 (sp3 C-H), 1709 (C = O ester), 1603 (C = C aromatic), 1132 (C-O, ester); ^1^H NMR (DMSO-*d*_6_, δ ppm): 7.03 (d, *J* = 8.0 Hz, 1H, H-6′), 6.97 (d, *J* = 1.6 Hz, 1H, H-4), 6.89 (d, *J* = 2.4 Hz, 2H, H-2, H-6), 6.79 (d, *J* = 1.6 Hz, 1H, H-3′), 6.49 (dd, *J* = 2.4, 2.0 Hz, 1H, H-5′), 5.99 (m, 1H. H-8′), 5.15 (s, 2H, -CH_2_), 5.03 (m, 2H, H-9′), 3.77 (s, 3H, -OCH_3_), 3.36 (d, *J* = 7.6 Hz, 2H, H-7′); ^13^C NMR (DMSO-*d*_6,_ δ ppm); 166.7 (C = O ester), 165.6 (C = O ester), 159.1 (C-3, C-5), 150.9 (C-1′), 147.9 (C-2′), 145.1 (C-5′), 139.7 (C-2, C-6), 137.8 (C-4′), 130.9 (C-3′), 122.8 (C-6′), 120.9 (C-9′), 116.5 (C-4), 115.7 (C-8′), 113.0 (C-1), 61.3 (-OCH_3_), 56.2 (-CH_2_), 56.0 (C-7′).

**2-[2-methoxy-4-(prop-2-en-1-yl)phenoxy]-2-oxoethyl(2E)-3-(4-hydroxyphenyl)prop-2-enoate (6a)** solid; reaction time, 24 hr; yield, 82%; melting point, 114–116 °C; R_f_ 0.48 (n-hexane:ethyl acetate 2:1), FTIR ν_max_ cm^−1^: 3103 (-OH), 2956 (sp2 C-H), 2872 (sp3 C-H), 1726 (C = O), 1601 (C = C aromatic), 1122 (C-O, ester); ^1^H NMR (DMSO-*d*_6_, δ ppm): 10.08 (1H, s, -OH), 7.67 (d, *J* = 15.6 Hz, 1H, H-2), 7.59 (d, *J* = 8.4 Hz, 2H, H-2′, 6′), 7.01 (d, *J* = 8.0 Hz, 2H, H-3′, 5′), 6.95 (s, 1H, H-3″), 6.79 (d, *J* = 8.4 Hz, 1H, H-6″), 6.75 (d, *J* = 8.4 Hz, 1H, H-5″), 6.51 (d, *J* = 16.0 Hz, 1H, H-1), 5.98 (m, 1H. H-8″), 5.11 (m, 2H, H-9″), 5.02 (s, 2H, -CH_2_), 3.74 (s, 3H, -OCH_3_), 3.15 (d, *J* = 4.8 Hz, 2H, H-7′); ^13^C NMR (DMSO-*d*_6,_ δ ppm); 166.1 (C = O ester), 165.8 (C = O, ester), 150.6 (C-1″), 146.4 (C-4′), 144.8 (C-3), 143.9 (C-2″), 139.4 (C-5″), 137.8 (C-3′, C-5′), 137.3 (C-9″), 136.9 (C-1′), 136.5 (C-3″), 132.6 (C-4″), 131.9 (C-8″), 129.4 (C-2), 122.3 (C-6″), 120.7 (C-2′,C-6′), 60.4 (-OCH_3_), 56.9 (C-7″), 55.8 (-CH_2_).

**2-[2-methoxy-4-(prop-2-en-1-yl)phenoxy]-2-oxoethyl(2E)-3-(4-chloroyphenyl)prop-2-enoate (6b)** solid; reaction time, 24 hr; yield, 84%; melting point, 86–88 °C; R_f_ 0.52 (n-hexane:ethyl acetate 2:1), FTIR ν_max_ cm^−1^: 3083 (sp2 C-H), 2878 (sp3 C-H), 1716 (C = O ester), 1615 (C = C aromatic), 1158 (C-O, ester); ^1^H NMR (DMSO-*d*_6_, δ ppm): 7.75 (d, *J* = 16.0 Hz, 1H, H-2), 7.40 (d, *J* = 4.8 Hz, 2H, H-2′, 6′), 7.37 (d, *J* = 4.8 Hz, 2H, H-3′, 5′), 7.01 (d, *J* = 8.0 Hz, 1H, H-6″), 6.87 (dd, *J* = 8.8, 1.6 Hz, 1H, H-5″), 6.81 (d, *J* = 2.0 Hz, 1H, H-3″), 6.52 (d, *J* = 16.0 Hz, 1H, H-1), 5.96 (m, 1H. H-8″), 5.11 (m, 2H, H-9″), 5.06 (s, 2H, -CH_2_), 3.84 (s, 3H, -OCH_3_), 3.35 (d, *J* = 6.8 Hz, 2H, H-7′); ^13^C NMR (DMSO-*d*_6,_ δ ppm); 166.1 (C = O ester), 165.8 (C = O, ester), 150.6 (C-1″), 146.4 (C-4′), 144.8 (C-3), 143.9 (C-2″), 139.4 (C-5″), 137.8 (C-3′, C-5′), 136.5 (C-9″), 131.9 (C-1′), 129.2 (C-3″), 120.7 (C-4″), 117.3 (C-8″), 116.2 (C-2), 115.5 (C-6″), 114.2 (C-2′,C-6′), 60.4 (-OCH_3_), 55.8 (C-7″), 40.7 (-CH_2_).

### Atmospheric pressure plasma jet (APPJ) and plasma treatment condition

The used plasma device mainly consists of electrodes, dielectrics, and a high-voltage power supply (Fig. 2(a)). The voltage and current graph of APPJ was shown in Fig. 2(b), in which the RMS voltage is 1.1 kV, RMS current is 30 mA, the frequency is 22 kHz and waveforms have a profile with an average power of 3 W. The N_2_ gas with the flow rate of 1lpm (litter per minute) was used and a plasma plume was ejected into the open air through a hole at distal end of plasma device. The OES spectra of APPJ emission was recorded by using HR4000CG-UV-NIR (Ocean Optics, FL, USA) and optical fiber (QP400-2-SR) with a diameter of 400 mm. Fig. 2(c) presents the optical emission spectrum (OES) (for more clarity, x-axis of OES spectrum is broken and presented in Figure S1(a–c).

We treated eugenol derivatives (ED) with APPJ as shown in Fig. 2(a). For plasma treatment, 1 ml of ED compounds (20 mg/ml concentration in DMSO) were placed in 24 well plate (liquid sample height was measured around 4.6 mm from bottom of well plate to the top of liquid surface) and then treated with plasma by maintaining the distance 9 mm from the end of nozzle to the liquid sample surface. The distance between the end of active plasma and liquid sample surface was recorded around 5 mm. Plasma treatment produced gentle movement in the liquid ED sample and caused slight reduction in sample volume which was fixed immediately after treatment to maintain 1 ml sample volume however, no significant change in temperature was observed during treatment.

### Cell culture conditions

Murine melanoma (B16F10) and murine skin fibroblast (L929) cells were obtained from American Type Culture Collection (ATCC), while human skin keratinocyte cells (HaCaT) were obtained from Yosei University, Seoul, Korea. All cell lines were cultured in Dulbecco’s modified Eagle’s medium (DMEM; WEL GENE) and supplemented with 10% fetal bovine serum (FBS; biowest) in a humidified incubator containing 5% CO_2_ at 37 °C throughout the experiment.

### Cytotoxicity and cell morphology

To test the cytotoxicity, an MTT assay (3-(4,5-Dimethylthiazol-2-yl)-2,5-Diphenyltetrazolium Bromide) was performed^55^ using two healthy and one cancer cell lines. The healthy cell lines were immortalized murine skin fibroblasts (L929) and human skin keratinocytes (HaCaT), while the cancer cell line was murine melanoma (B16F10) cells. All 3 types of cells (1.5 × 10^5^ cells/ml) were cultured in 24-well plates (SPL Korea) with complete DMEM medium at 37 °C (5% CO_2_ atmosphere) before being exposed to the various concentrations of test compounds. The eugenol derivatives (4a-4e, 6a and 6b) were dissolved in DMSO and diluted with DMEM medium to achieve the final concentrations of 125, 62.5, 31.25, and 15.62 μg/ml, and were then added to the cells following 24 hr incubation. The MTT assay was then conducted, absorbance was measured at 570 nm wavelength, and the results were calculated as percentages of the control, the untreated sample.

For cell morphology, the melanoma cells were seeded in cell culture plates (SPL Korea) and incubated at 37 °C (5% CO_2_ atmosphere). The cells were then incubated with the various concentrations of 4b and 6a (0 (con), 14, 21, 28, 35, and 42 μg/ml) following 24 hr incubation. The cell morphology was accessed and images were captured using an inverted fluorescent microscope (Nikon, ECLIPSE, Ti).

### Melanin content measurement

Melanoma (1.5 × 10^5^) cells were seeded in cell culture plates and allowed to attach for 24 hr. Then each eugenol derivative was added and tested separately for melanin activation after 24 hr of incubation. After incubation, cells were detached using trypsin/EDTA and centrifuged at 1000 rpm for 5 min to collect the cell pellets. The cell pellets were solubilized in 1N NaOH at 60 °C for 1 hr. The melanin content was measured spectrophotometrically at 405 nm wavelength using a microplate reader (BioTek, synergy HT)^56^. For further investigation, a non-thermal atmospheric pressure plasma jet was used to treat the most active ED, 4b and 6a for 3, 5, and 10 min. Plasma-treated and non-plasma-treated ED (4b and 6a) were added to the cells, pre-incubated with or without α-MSH (100 nM) for a further 24 hr and melanin content was assayed as described above. Kojic acid can inhibit and α-MSH can stimulate the melanin in the cells, therefore we used them as negative and positive controls.

### Intracellular ROS and cAMP measurements

Intracellular reactive oxygen species (ROS) were quantified using carboxy-H_2_DCFDA (5-(and 6)-carboxy-20,70-dichlorodihydrofluorescein diacetate), by monitoring the fluorescence intensity^57^.

Briefly, various concentrations of as-synthesized 4b and 6a or plasma-treated 4b and 6a were incubated with cells. After 24 hr, the cells were centrifuged for 5 min at 1000 rpm and the collected cell pellet was washed with PBS and labeled with carboxy-H_2_DCFDA dye following 30 min of incubation at 37 °C. The samples were centrifuged again and each pellet was mixed with 500 μl PBS. The 200 μl samples were then transferred in 96-well plates (black plate, clear bottom, corning incorporated USA) and fluorescence was measured at excitation/emission: 485/528 nm using a microplate reader.

For cAMP measurement, the 4b and 6a were treated with plasma for 3, 5, and 10 min, and then added to the cells, pre-incubated with or without α-MSH (100 nM) for 24 hr. The cells were trypsinised and centrifuged to obtain the cell pellets. The cell pellet was lysed and the cAMP level was measured using a cAMP Kit (Abcam, USA) according to the manufacturer’s instructions.

### Western blot analysis

Melanoma cells were incubated with as-synthesized and plasma-treated 4b and 6a test compounds for 24 hr. Following incubation, treated and untreated cells were lysed in RIPA buffer (Cell Signaling Technology, USA), whole cell protein was extracted and subjected to electrophoresis in 12% SDS-PAGE, and blotted onto nitrocellulose membranes. The membrane was probed with the antibodies raised against MITF, tyrosinase and TRP1 (Abcam, USA). The bands were detected using Super Signal West Pico Chemiluminescent Substrate (Pierce, Rockford, IL, USA) and images were taken using a Vilver imaging system (Vilver, Upland, CA, USA).

### Mushroom tyrosinase assay protocol

The mushroom tyrosinase was used for *in vitro* bioassays as described previously with some modifications^58^,^59^. Briefly, 140 μl of phosphate buffer (20 mM, pH 6.8), 20 μl of mushroom tyrosinase (30 U/ml), and 20 μl of the inhibitor solution were placed in the wells of a 96-well plate. After pre-incubation for 10 min at room temperature, 20 μl of L-DOPA (3,4-dihydroxyphenylalanine) (0.85 mM) was added and the plate was further incubated at 25 °C for 20 min. Subsequently the absorbance of dopachrome was measured at 492 nm using a micro plate reader (OPTI Max, Tunable). Kojic acid was used as a reference inhibitor. The extent of inhibition by the test compounds was expressed as the percentage of concentration necessary to achieve 50% inhibition (IC_50_). Each concentration was analyzed in three independent experiments run in triplicate. The IC_50_ values were determined by the data analysis and graphing software, Origin.

### Kinetic analysis of the inhibition of mushroom tyrosinase

A series of experiments were performed to determine the inhibition kinetics using the following method^60^,^61^. The inhibitor (6a) concentrations 0, 6.7, 13.5, 27 μM for Lineweaver-Burk plot and 0, 1.7, 3.4, 6.7, 13.5, 27, 54 μM for Dixon plot were used. Inhibitor (4b) concentrations 0, 18, 36.5, 73, 146 μM for Lineweaver-Burk plot and 0, 9, 18, 36.5, 73, 146 μM for Dixon plot were used, respectively. The substrate L-DOPA concentration was between 0.0625 and 2 mM in all kinetic studies. Pre-incubation and measurement times were the same, as discussed for the mushroom tyrosinase inhibition assay protocol. The formation of DOPAchrome was continuously monitored at 475 nm for 5 min at a 30 sec intervals in the microplate reader after the addition of the enzyme. The inhibition type on the enzyme was assayed by Lineweaver–Burk plots of inverse of velocities (1/V) versus the inverse of the substrate concentration 1/[S] mM^-1^, and the inhibition constant K_i_ was determined by a Dixon plot of 1/V versus inhibitor concentrations.

## Molecular docking studies of synthesized compounds against mushroom tyrosinase

### Preparation of receptor.

A Molecular Operating Environment (MOE 2014) was used for the current studies^62^,^63^. The protein preparation steps involved 3D protonation, energy minimization, and active site identification. The crystal structure of the mushroom tyrosinase was obtained from a protein data bank with co-crystallized ligand^36^. Water molecules were removed. Protein was energy minimized and 3D protonated by using the structure preparation module of MOE. Since the protein contains the co-crystallized ligand, the active site was identified around the co-crystallized ligand by using the LigX module of the MOE. The pocket was found to be a deep cavity lined with the key residues including both hydrophobic and hydrophilic amino acids. The ligand files for the molecular docking studies were prepared in Molecular Operating Environment (MOE-2014) by Chemical Computing Group (CCG)^36^ and were followed by energy optimization at a standard MMFF94 force field level, with a 0.0001 kcal/mol energy gradient convergence criterion^64^. The optimized geometries were saved in a molecular data base file for further studies.

The optimized ligands were docked with the mushroom tyrosinase (PDB code: 2Y9X) protein^33^,^36^ using the MOE-Dock program. Thirty independent docking runs were performed using the MOE docking simulation program. The docked poses were analyzed and the best scored pose for each compound was chosen for further studies of interaction evaluation. The 2D ligand-protein interactions were visualized using the MOE ligand interactions program.

### CD spectrum analysis

For the CD spectroscopic studies^65^,^66^,^67^ a J-815 spectrophotometer (Jasco, Japan) was used, equipped with a Peltier system that controlled the temperature. (1S)-(+)-10-camphorsulfonic acid (Aldrich, Milwaukee, WI) with a molar extinction coefficient of 34.5 M/cm at 285 nm and a 2.36 M/cm molar ellipticity (θ) at 295 nm was used for the CD calibrations. The samples were pre-equilibrated at the desired temperature for 15 min, and the scan speed was fixed for adaptative sampling (error F 0.01) with a response time of 1 sec and a 1 nm bandwidth. The secondary structure of the mushroom tyrosinase was monitored by using a 1.0 cm path length cuvette. The mushroom tyrosinase (0.2 mg/ml) was prepared in phosphate buffer (20 mM) at pH 6.8 for CD analysis. We used the 0.1 mM concentration of all derivatives in 1 ml buffer solution, with or without 10 min plasma treatment. All spectra were collected in triplicate from 200 to 250 nm and the background was corrected against the buffer (blank). The percentages of secondary structures of proteins were calculated using inbuilt Jasco software.

### Statistical analysis

All values are represented as mean ± SD of the indicated number of replicates. The statistical analyses of the data were performed using Student’s t-test to establish the significance between the data points, and significant differences are based on *p < 0.05, **p < 0.005.

## Additional Information

**How to cite this article**: Ali, A. *et al.* Influence of plasma-activated compounds on melanogenesis and tyrosinase activity. *Sci. Rep.* **6**, 21779; doi: 10.1038/srep21779 (2016).

## Supplementary Material

Supplementary Information

## Figures and Tables

**Table 1:** Tyrosinase inhibitory activity of test compounds before and after plasma treatment.

Samples	IC_50_(μg/ml)						
	**4a**	**4b**	**4c**	**4d**	**4e**	**6a**	**6b**
Con	25 ± 3	29 ± 5	15 ± 4	58 ± 7	19 ± 2	5 ± 2	7 ± 2
3 min	24 ± 3	24 ± 7	19 ± 5	47 ± 4	77 ± 7	5 ± 2	33 ± 5
5 min	20 ± 5	23 ± 2	24 ± 6	36 ± 3	72 ± 7	5 ± 2	40 ± 6
10 min	17 ± 1	14 ± 2	29 ± 4	29 ± 2	56 ± 3	3 ± 1	55 ± 5

IC_50_ for tyrosinase inhibition activity was determined for all test compounds (4a, 4b, 4c, 4d, 4e, 6a, and 6b) before and after plasma treatment (3, 5, and 10 min). All data were expressed as mean ± standard deviation.

**Table 2:** Effect of ED compounds and plasma treated ED compounds on the secondary structure of mushroom tyrosinase.

Samples	α-helix %	β-sheet %	Turn %	Random coil %
Tyrosinase enzyme alone	16	33	7	44
4a-0 min + Tyrosinase	15	35	5	45
4a-10 min + Tyrosinase	17	31	7	45
4b-0 min + Tyrosinase	16	29	10	45
4b-10 min + Tyrosinase	16	33	6	45
4c-0 min + Tyrosinase	16	35	4	45
4c-10 min + Tyrosinase	14	35	4	46
4d-0 min + Tyrosinase	15	33	7	45
4d-10 min + Tyrosinase	15	36	4	45
4e-0 min + Tyrosinase	15	33	6	46
4e-10 min + Tyrosinase	14	34	6	46
6a-0 min + Tyrosinase	20	25	11	44
6a-10 min + Tyrosinase	19	28	9	44
6b-0 min + Tyrosinase	15	37	3	45
6b-10 min + Tyrosinase	14	36	4	46
