# Supplementary material for: Influence of plasma-activated compounds on melanogenesis and tyrosinase activity
Source: Sci Rep. 2016 Mar 2;6:21779. doi: 10.1038/srep21779 (PMC4773869; doi:10.1038/srep21779)
Supplement: Supplementary Information [file srep21779-s1.pdf]

Supporting file

Influence of plasma-activated compounds on melanogenesis and tyrosinase activity

Anser Ali[1,2], Zaman Ashraf[3,4], Naresh Kumar[1,2], Muhammad Rafiq[3], Farukh Jabeen[6,7], Jihoon Park[5], Ki Hong Choi[5], SeungHyun Lee[1,2], Sung-Yum Seo[3], Eun Ha Choi[1,2,5]* and Pankaj Attri [2,5,8]*

[1]*Department of Plasma-Bio Display,* [2]*Plasma Bioscience Research Center, Kwangwoon University, 20 Kwangwoon-gil, Nowon-gu, Seoul 139-701, Republic of Korea*
[3]*Department of Biology, College of Natural Sciences, Kongju National University, Gongju 314-701, Republic of Korea*
[4]*Department of Chemistry, Allama Iqbal Open University, Islamabad 44000, Pakistan*
[5]*Department of Electrical and Biological Physics, Kwangwoon University, 20 Kwangwoon-gil, Nowon-gu, Seoul 139-701, Republic of Korea*
[6]*Florida Center of Heterocyclic Compounds, University of Florida, Gainesville, Fl, 32601, USA*
[7]*Center for Computationally Assisted Science and Technology, North Dakota State University, Fargo, ND, 58102, USA*
[8]*Graduate School of Information Science and Electrical Engineering, Kyushu University Fukuoka 819-0395, Japan*

Figure S1: Optical emission spectrum detected from atmospheric pressure plasma jet (APPJ) using HR4000 spectrometer. The x-axix of detected spectrum is broken as (a) 200-300 nm, (b) 300-500 nm and (c) 500-1100 nm wavelengths for more details and clarity of spectrum.

Figure S2: NMR before and after the plasma treatment to (a) 4b and (b) 6a compounds.

Figure S3: 2D interactions of eugenol derivatives. 2D interactions of as-synthesized eugenol derivatives (a) 4a, (b) 4b, (c) 4c, (d) 4d, (e) 4e, (f) 6a, (g) 6b, and (h) a reference compound kojic acid are given. However, the 2D image (i) shows plasma treated 4b and image (j) shows plasma treated 6a interactions. (color and pattern details are given in the legend).

Figure S4: Cell viability measurement. The murine melanoma (B16F10), human skin keratinocyte (HaCaT), and murine skin fibroblast (L929) cells were incubated with different concentrations of (a) 4a, (b) 4c, (c) 4d, (d) 4e, and (e) 6b ED compounds and cell viability was accessed by MTT assay. The data were expressed as a percentage of the control (normalized to

100%) from three independent experiments with mean ± standard deviation and were analyzed using Student's t-tests, *p<0.05, **p<0.005.

Figure S5: Cell morphology. Melanoma cells were incubated with various concentrations of 4b (a1) 0 μg/ml (control), (a2) 14 μg/ml, (a3) 21 μg/ml, (a4) 28 μg/ml, (a5) 35 μg/ml, (a6) 42 μg/ml and 6a (b1) 0 μg/ml (control), (b2) 14 μg/ml, (b3) 21 μg/ml, (b4) 28 μg/ml, (b5) 35 μg/ml, (b6) 42 μg/ml for 24 hours and images with a 100-μm scale bar were taken by inverted fluorescent microscope.

Figure S6: Intracellular melanin measurement. B16F10 cells were incubated with various concentrations of (a) 4a, (b) 4c, (c) 4d, (d) 4e, and (e) 6b and intracellular melanin was detected. All data were expressed as a percentage of the control (normalized to 100%) from three independent experiments with mean ± standard deviation and were analyzed using Student's t-tests, *p<0.05.

Figure S7. Images of melanoma B16F10 cell pallets. B16F10 cells were incubated with indicated concentrations of 4b and 6a for 24 hr and then centrifuged to photograph the cell pellet. The cells grown with media only are shown as the control, while the cells grown with α-MSH and Kojic acid in media are shown as positive and negative controls, respectively.

Table S1: Representation of binding affinity with specific interactions of as-synthesized eugenol derivatives (a) 4a, 4b, (b) 4c, 4d, (c) 4e, 6a, (d) 6b, kojic acid (a reference compound) is given. However, table (e) shows interactions for plasma treated 4b and plasma treated 6a eugenol derivatives.

<table>
<tr><th rowspan="2">Codes</th><th colspan="2">Binding Energy (kcal/mol)</th><th colspan="2">H-Bonding</th><th rowspan="2">π-H interaction</th><th rowspan="2">π-π interaction</th><th rowspan="2">Hydrophobic Interactions</th><th rowspan="2">Polar interaction</th><th rowspan="2">Cu-ligand Interaction</th></tr>
<tr><th>S</th><th>London dG</th><th>Interacting residues</th><th>Distance (Å)</th></tr>
<tr><td rowspan="2">4a</td><td>-5.8997</td><td>-9.5568</td><td>Arg268-OH</td><td>3.0</td><td>-</td><td>Val283</td><td>Val283
Phe264
Val248</td><td>His244
His263
Glu322
Arg2608</td><td></td></tr>
<tr><td colspan="9">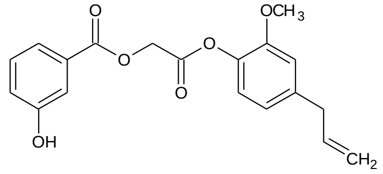
2-[2-methoxy-4-(prop-2-en-1-yl)phenoxy]-2-oxoethyl 3-hydroxybenzoate (4a)</td></tr>
<tr><td rowspan="2">4b</td><td>-6.4590</td><td>-8.8773</td><td>His263</td><td>3.9</td><td>His85</td><td>-</td><td>Phe264
Val283
Val248</td><td>Asn260
His244
His85
Asn81
Glu322</td><td>CU400-C=O</td></tr>
<tr><td colspan="9">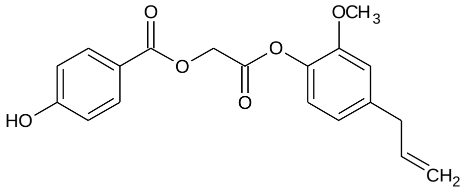
2-[2-methoxy-4-(prop-2-en-1-yl)phenoxy]-2-oxoethyl 4-hydroxybenzoate (4b)</td></tr>
</table>

Table S1(a)

Codes	Binding Energy (kcal/mol)		H-Bonding		π-H interaction	π-π interaction	Hydrophobic Interactions	Polar interaction	Cu-ligand Interaction
	S	London dG	Interacting residues	Distance (Å)					
4c	-6.0058	-7.8342	-	-	-	-	Val283<br>Phe264<br>Val248	His244<br>Glu322<br>Asn81<br>Asn260	
	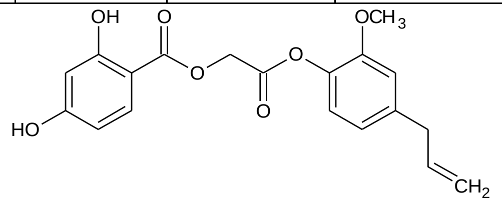<br>2-[2-methoxy-4-(prop-2-en-1-yl)phenoxy]-2-oxoethyl 2,4-dihydroxybenzoate (4c)								
4d	-5.7489	-8.5462	-	-	-	His263	Val283<br>Phe264<br>Val248	His263<br>His85<br>His244<br>Glu322<br>Asn260	
	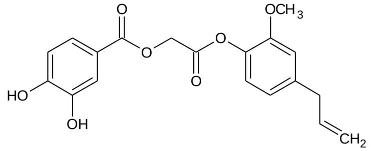<br>2-[2-methoxy-4-(prop-2-en-1-yl)phenoxy]-2-oxoethyl 3,4-dihydroxybenzoate (4d)								

Table S1(b)

Codes	Binding Energy (kcal/mol)		H-Bonding		π-H interaction	π-π interaction	Hydrophobic Interactions	Polar interaction	Cu-ligand Interaction
	S	London dG	Interacting residues	Distance (Å)					
4e	-6.1226	-9.2364	-	-	-	-	Val283<br>Ala264<br>Val248<br>Phe264	His85<br>His244<br>Asn260	
	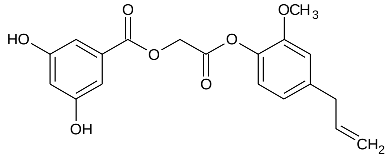<br>2-[2-methoxy-4-(prop-2-en-1-yl)phenoxy]-2-oxoethyl 3,5-dihydroxybenzoate (4e)								
6a	-6.5807	-8.7758	Glu322-OH	3.81	His85<br>Val283	His263	Val248<br>Phe264<br>Val283	His85<br>His244<br>Glu322<br>Asn260<br>His263	CU-400-C=O
	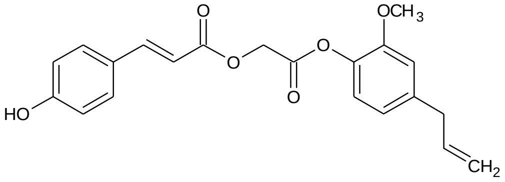<br>2-[2-methoxy-4-(prop-2-en-1-yl)phenoxy]-2-oxoethyl(2E)-3-(4-hydroxyphenyl)prop-2-enoate (6a)								

Table S1(c)

Codes	Binding Energy (kcal/mol)		H-Bonding		π-H interaction	π-π interaction	Hydrophobic Interactions	Polar interaction	Cu-ligand Interaction
	S	London dG	Interacting residues	Distance (Å)					
6b	-5.5801	-9.0163	-	-	His85	-	Val283<br>Phe264<br>Val248	His85<br>Glu322<br>Asn260	
	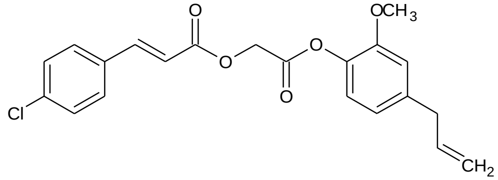<br>2-[2-methoxy-4-(prop-2-en-1-yl)phenoxy]-2-oxoethyl(2E)-3-(4-chloroyphenyl)prop-2-enoate (6b)								
Kojic acid	-5.0116	-8.3519	His85	2.83	-	-	Val283	His85<br>Asn260<br>His263	Cu-C=O<br>Cu-O-H
	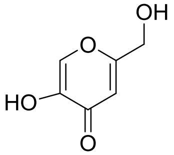<br>Kojic acid								

Table S1(d)

Codes	Binding Energy (kcal/mol)		H-Bonding		π-H interaction	π-π interaction	Hydrophobic Interactions	Polar interaction	Cu-ligand Interaction
	S	London dG	Interacting residues	Distance (Å)					
Plasma treated 4b	-6.8072	-9.9701	Ala-246<br>Glu-322<br>Ala-323<br>His-263	2.89<br>3.41<br>2.97<br>3.63	His-85<br>Val-283	-	Phe264<br>Val248<br>Met257	His85<br>His244<br>His263<br>Glu322<br>Asn260<br>His81	Cu-C=O
	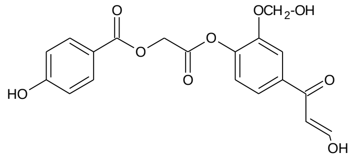<br>Proposed structure for plasma treated 4b								
Plasma treated 6a	-6.8403	-9.1881	-	-	His-85	-	Val248<br>Phe264<br>Val283	Thr261<br>His244<br>Glu322<br>Asn260<br>His85<br>His263<br>Asn-85	Cu-C=O
	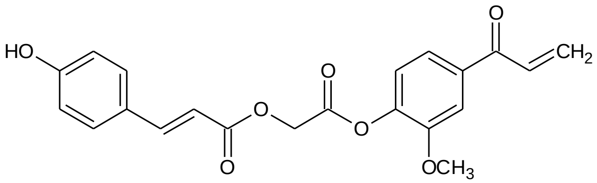<br>Proposed structure for plasma treated 6a								

Table S1(e)

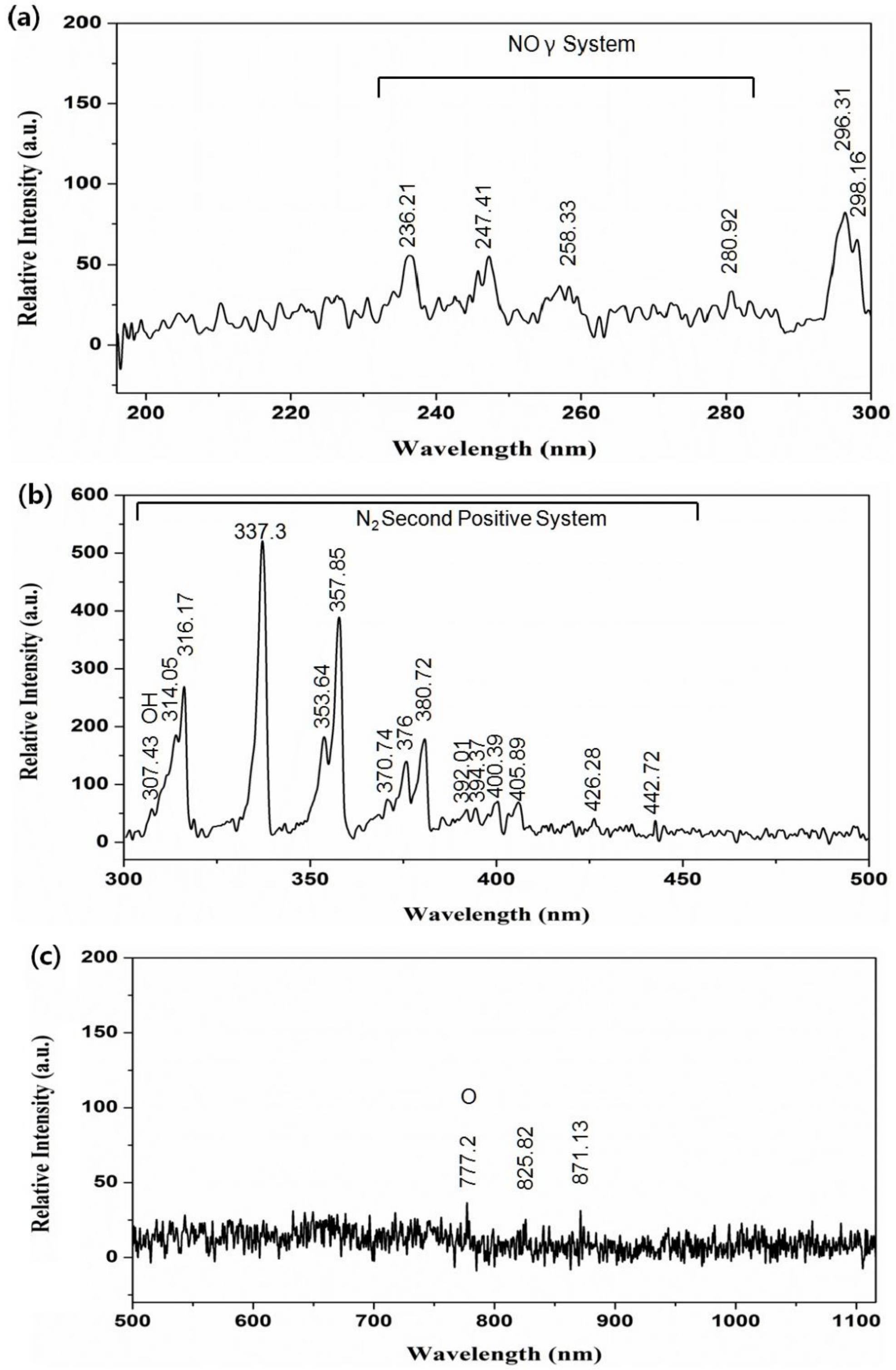
(a)
NO γ System
236.21
247.41
258.33
280.92
296.31
298.16
Relative Intensity (a.u.)
Wavelength (nm)
(b)
N2 Second Positive System
337.3
357.85
316.17
314.05
OH
307.43
353.64
370.74
376
380.72
392.01
394.37
400.39
405.89
426.28
442.72
Relative Intensity (a.u.)
Wavelength (nm)
(c)
O
777.2
825.82
871.13
Relative Intensity (a.u.)
Wavelength (nm)

Figure S1

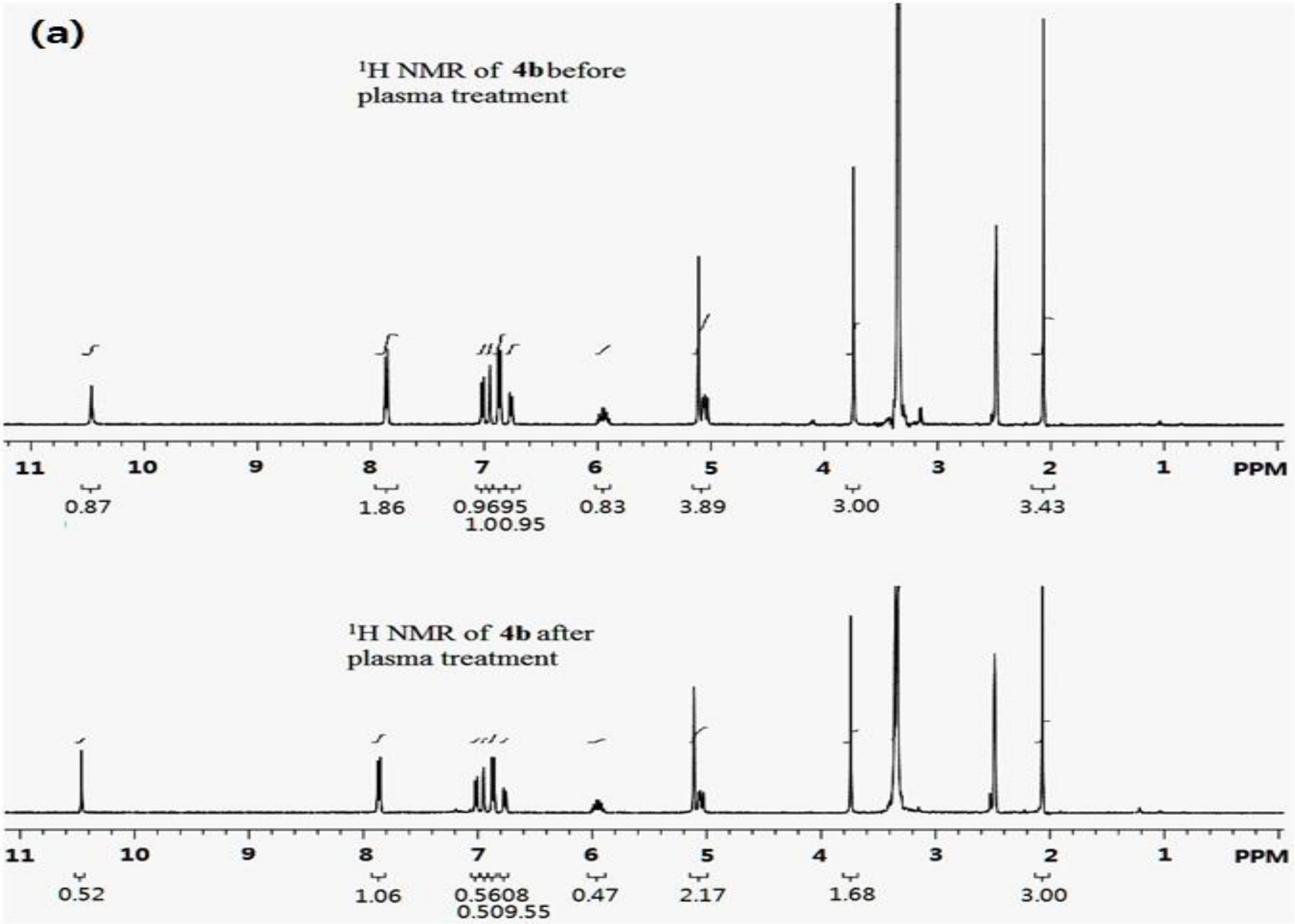

Figure S2 (continue)

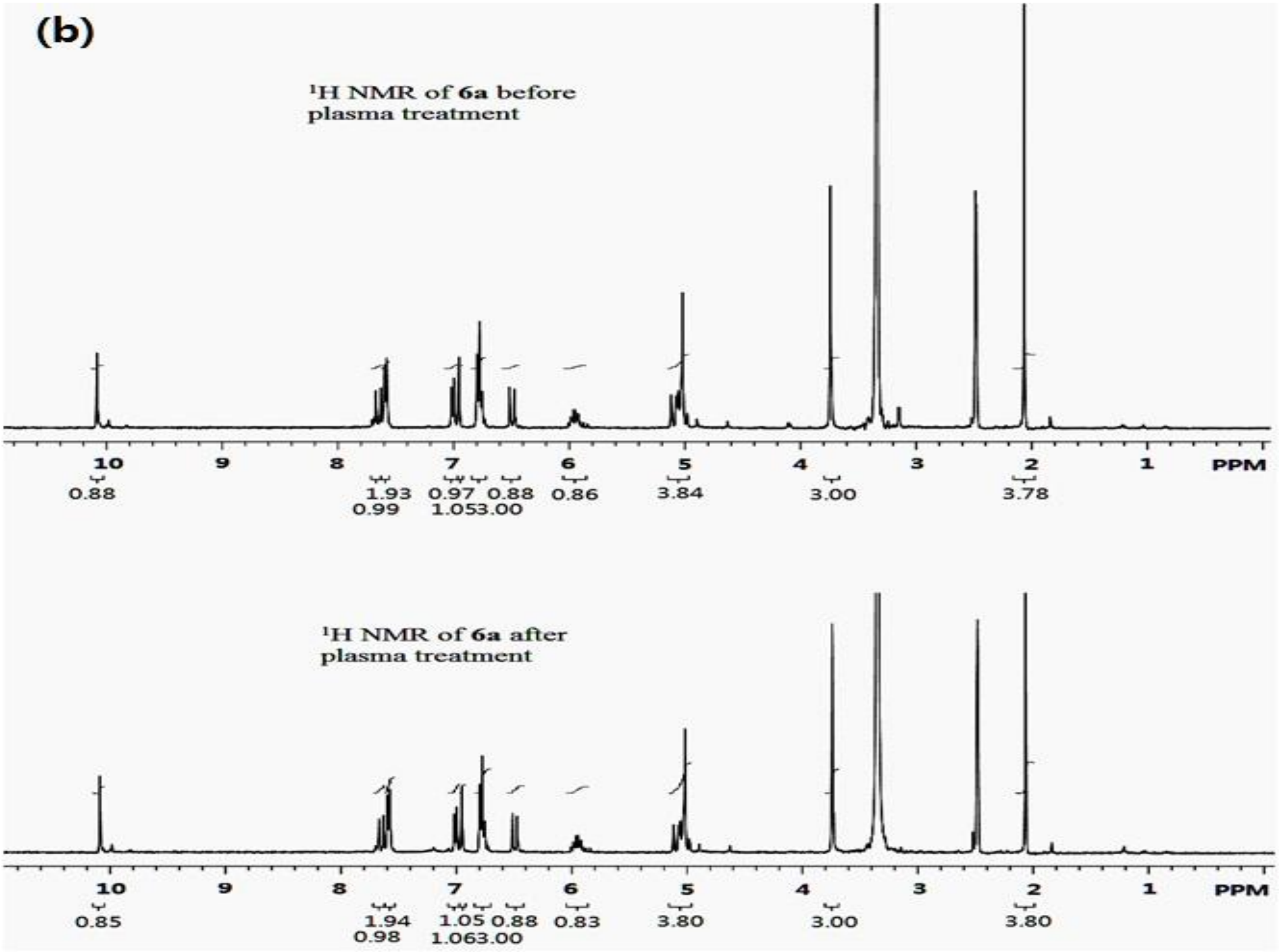
(b)
¹H NMR of 6a before
plasma treatment
10
9
8
7
6
5
4
3
2
1
PPM
0.88
1.93
0.99
0.97
1.05
3.00
0.88
0.86
3.84
3.00
3.78
¹H NMR of 6a after
plasma treatment
10
9
8
7
6
5
4
3
2
1
PPM
0.85
1.94
0.98
1.05
1.06
3.00
0.88
0.83
3.80
3.00
3.80

Figure S2

(a)

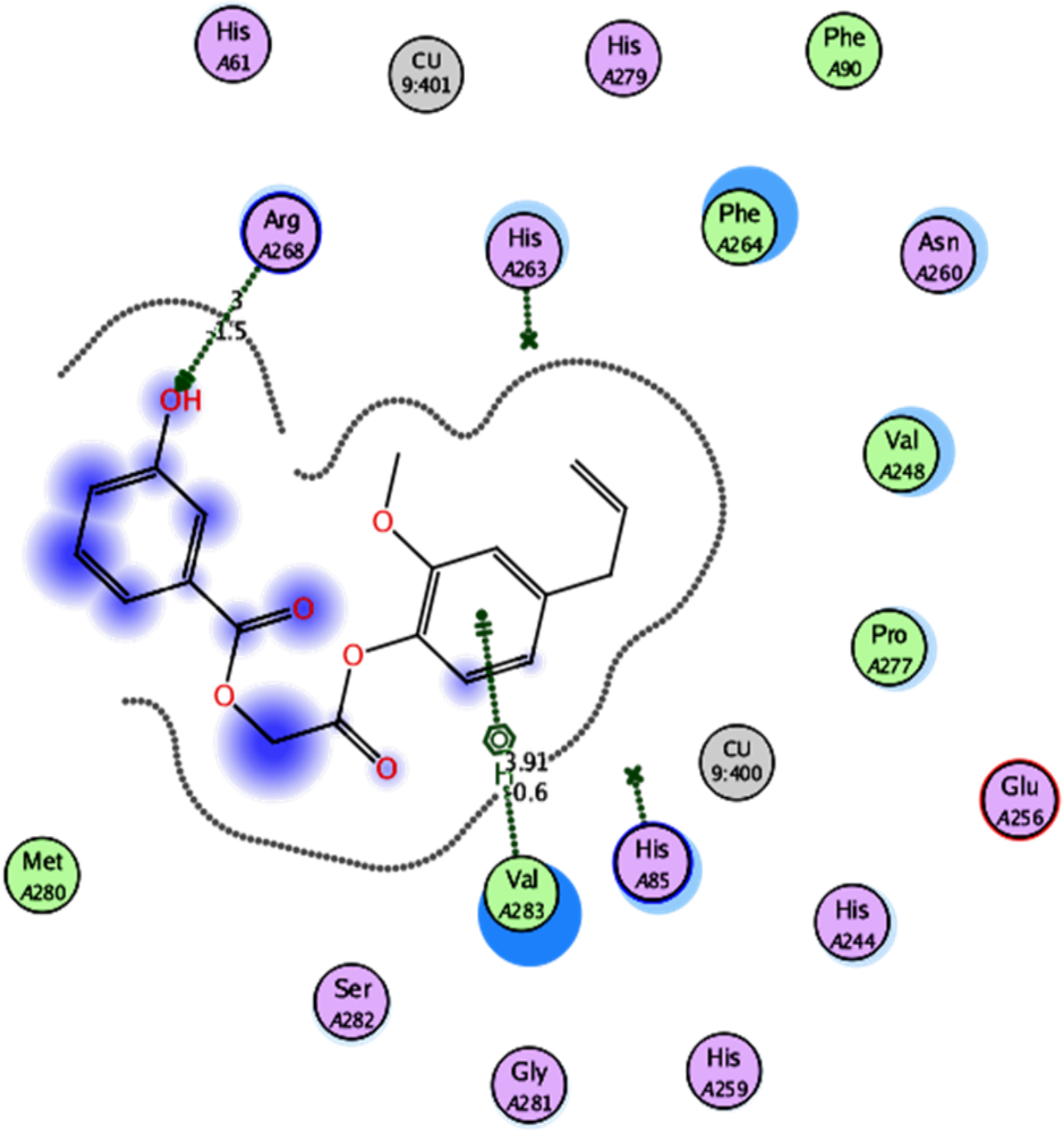

Figure S3 (continue)

(b)

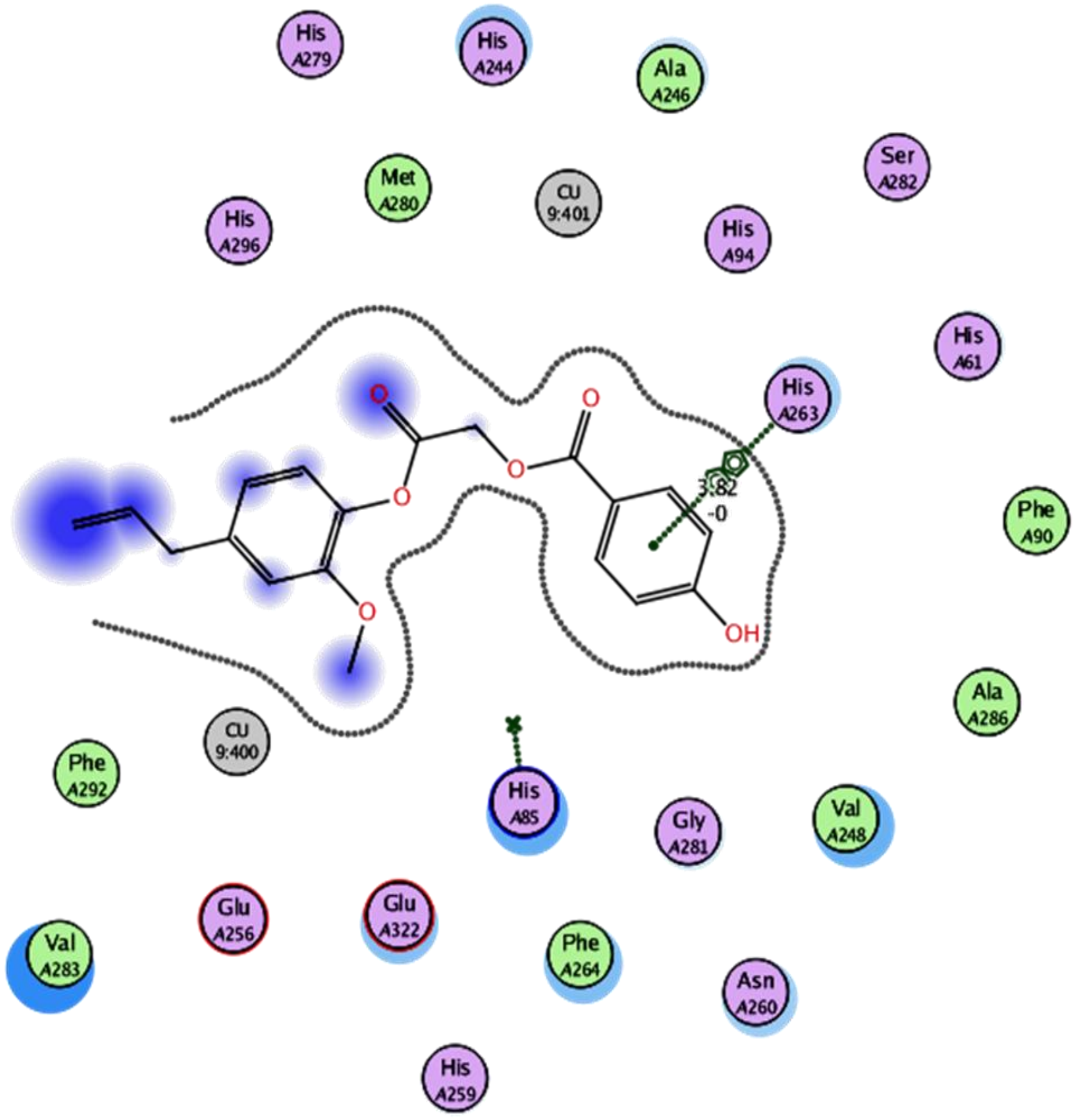

Figure S3 (continue)

(c)

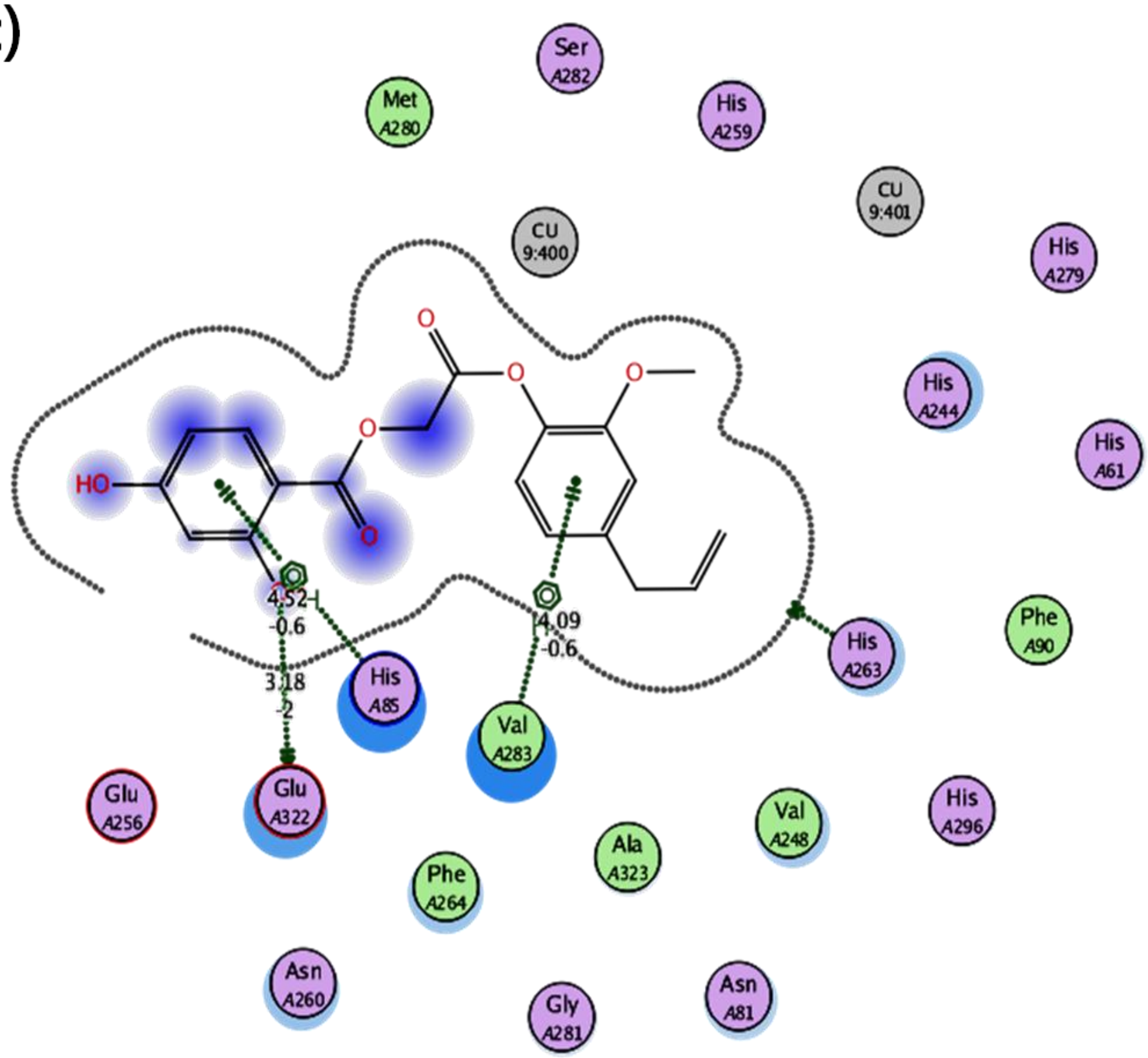

Figure S3 (continue)

(d)

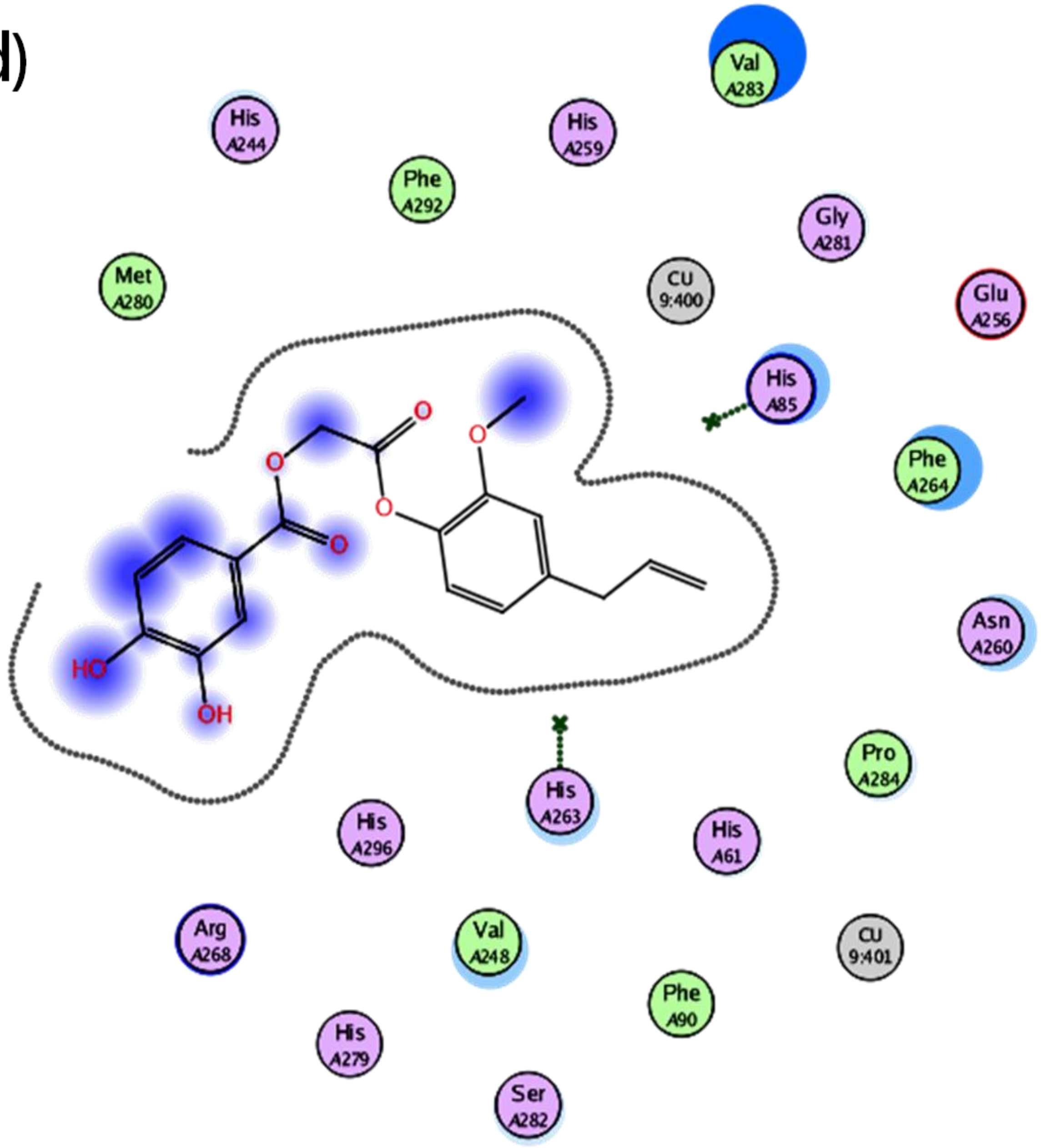

Figure S3 (continue)

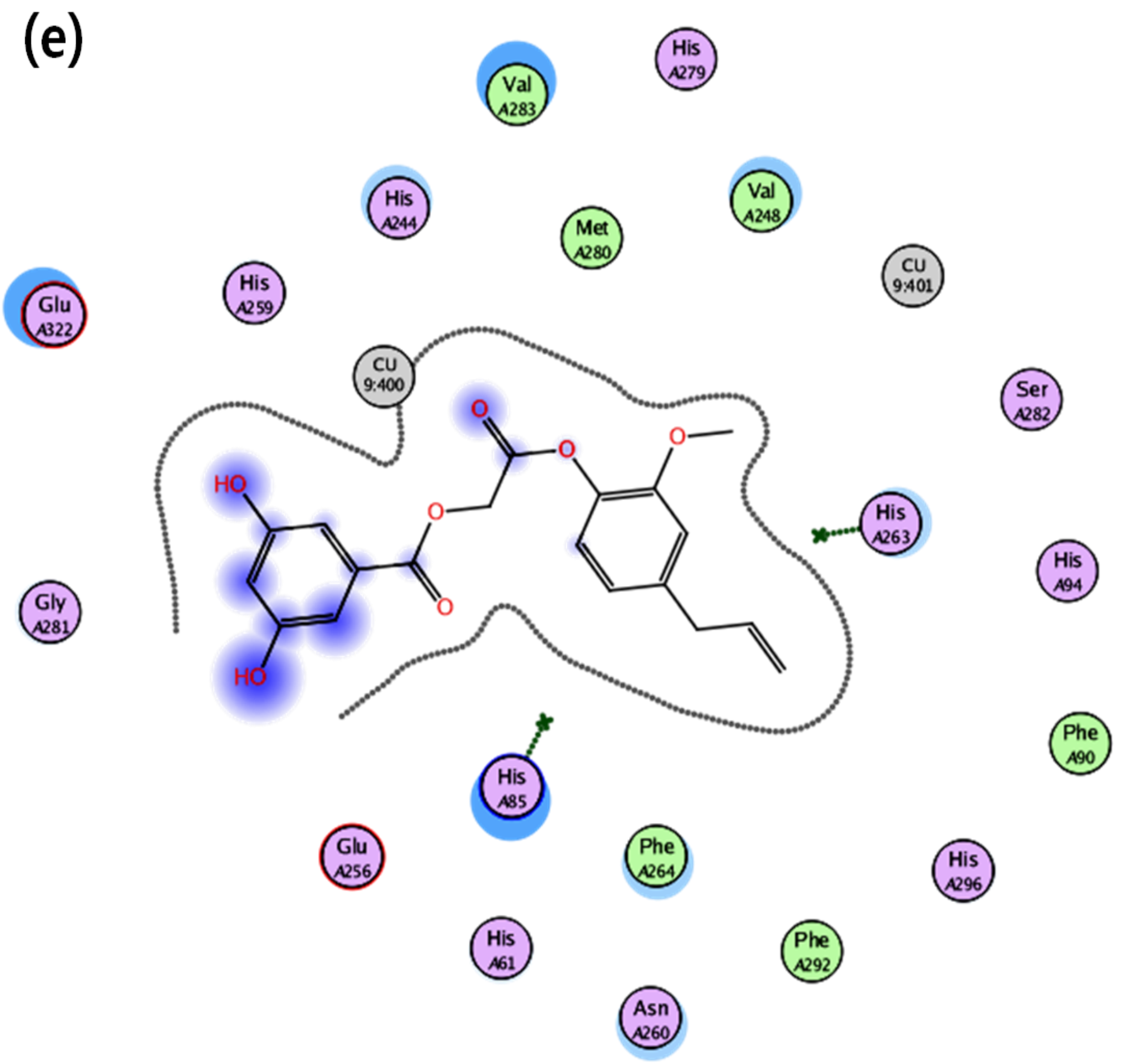

Figure S3 (continue)

(f)

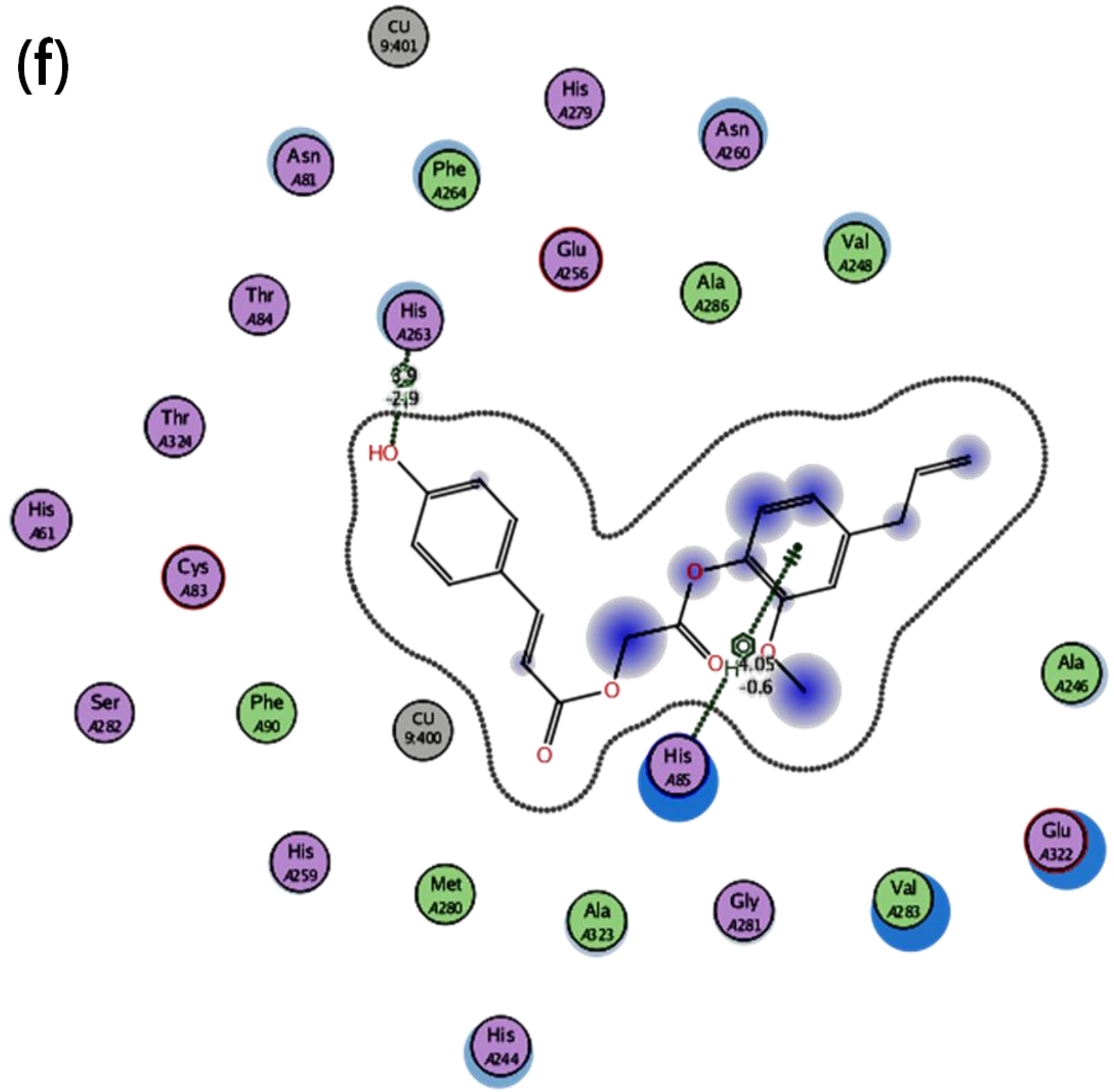

Figure S3 (continue)

(g)

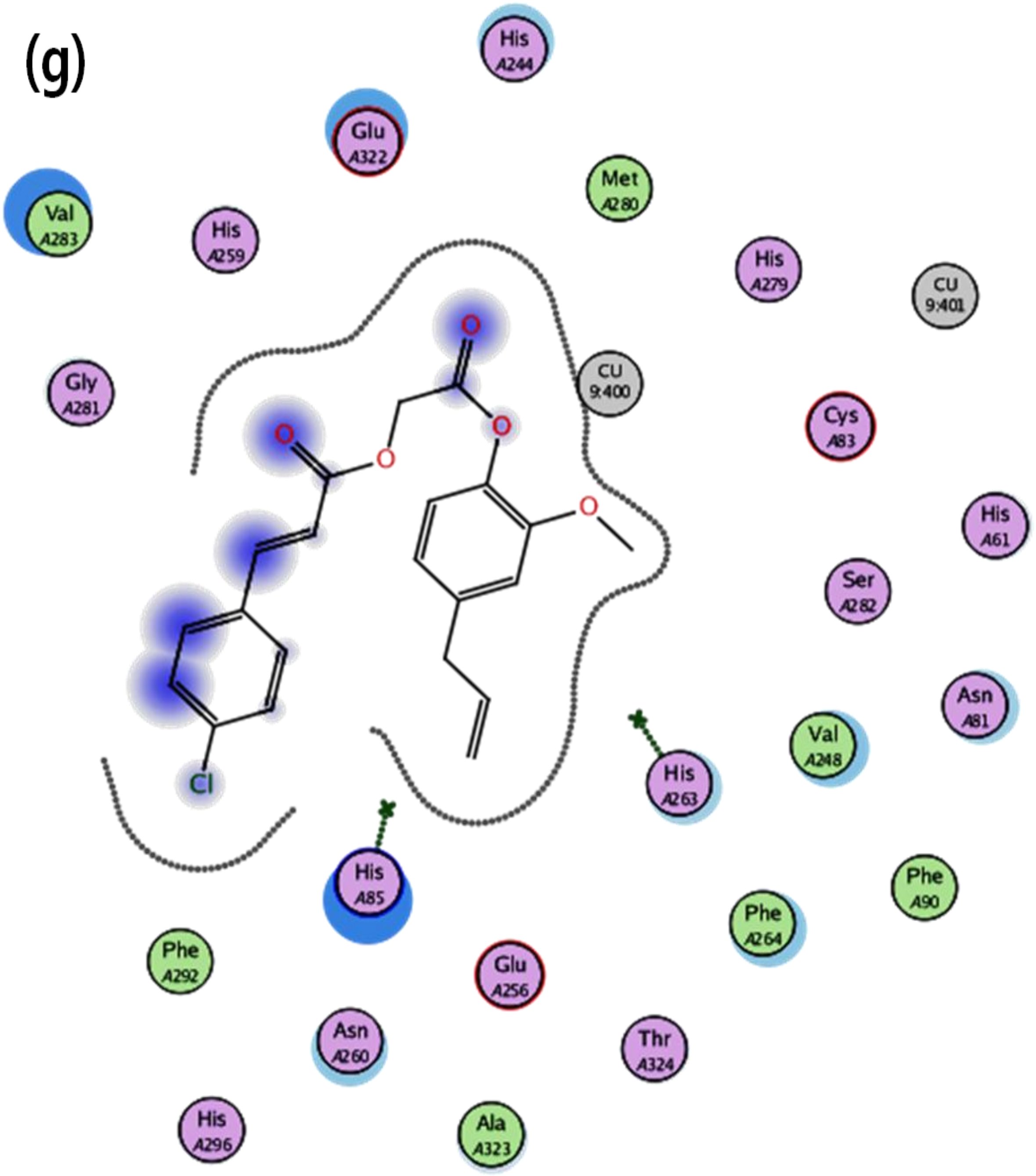

Figure S3 (continue)

(h)

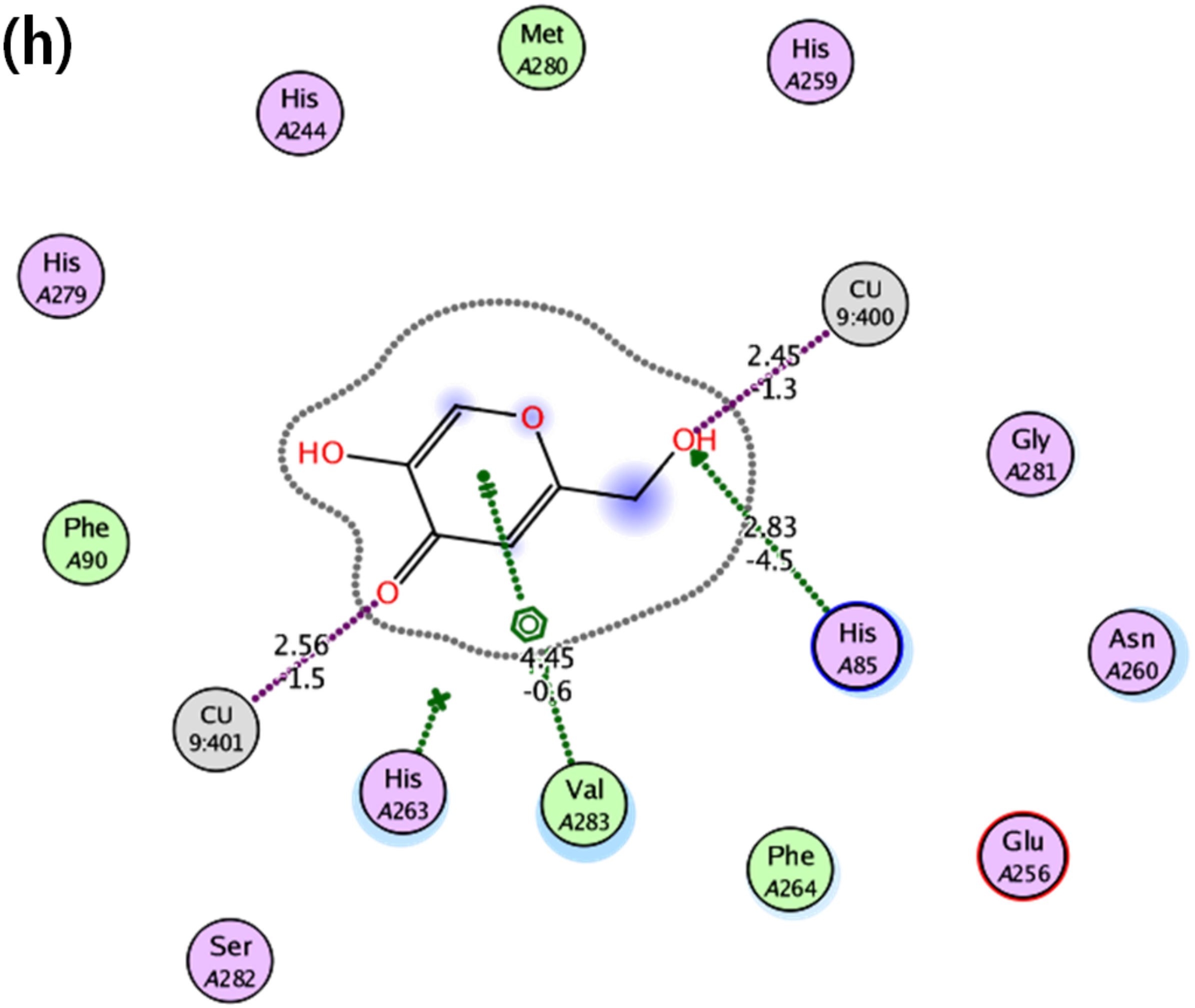

Figure S3 (continue)

(i)

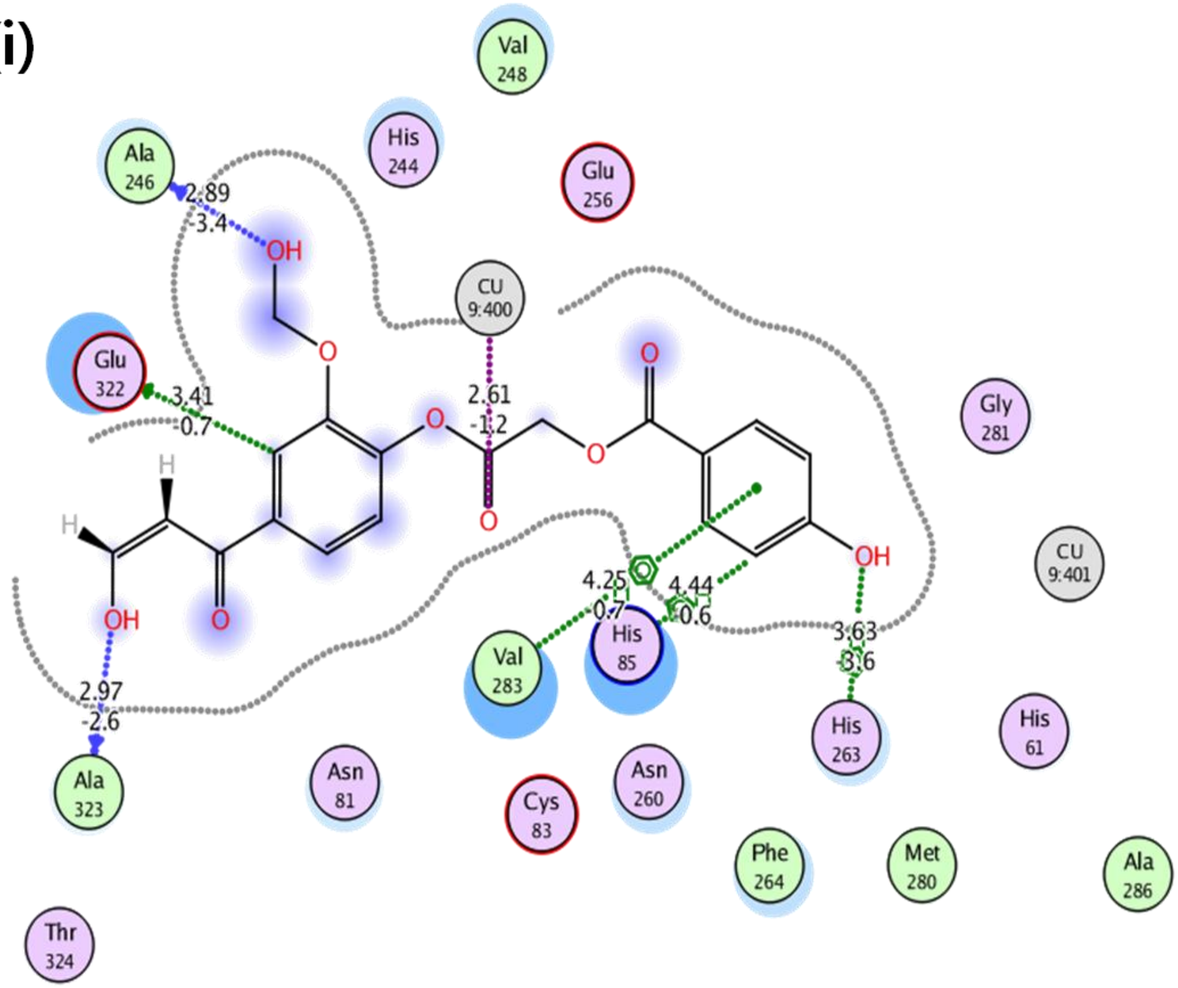

Figure S3 (continue)

(j)

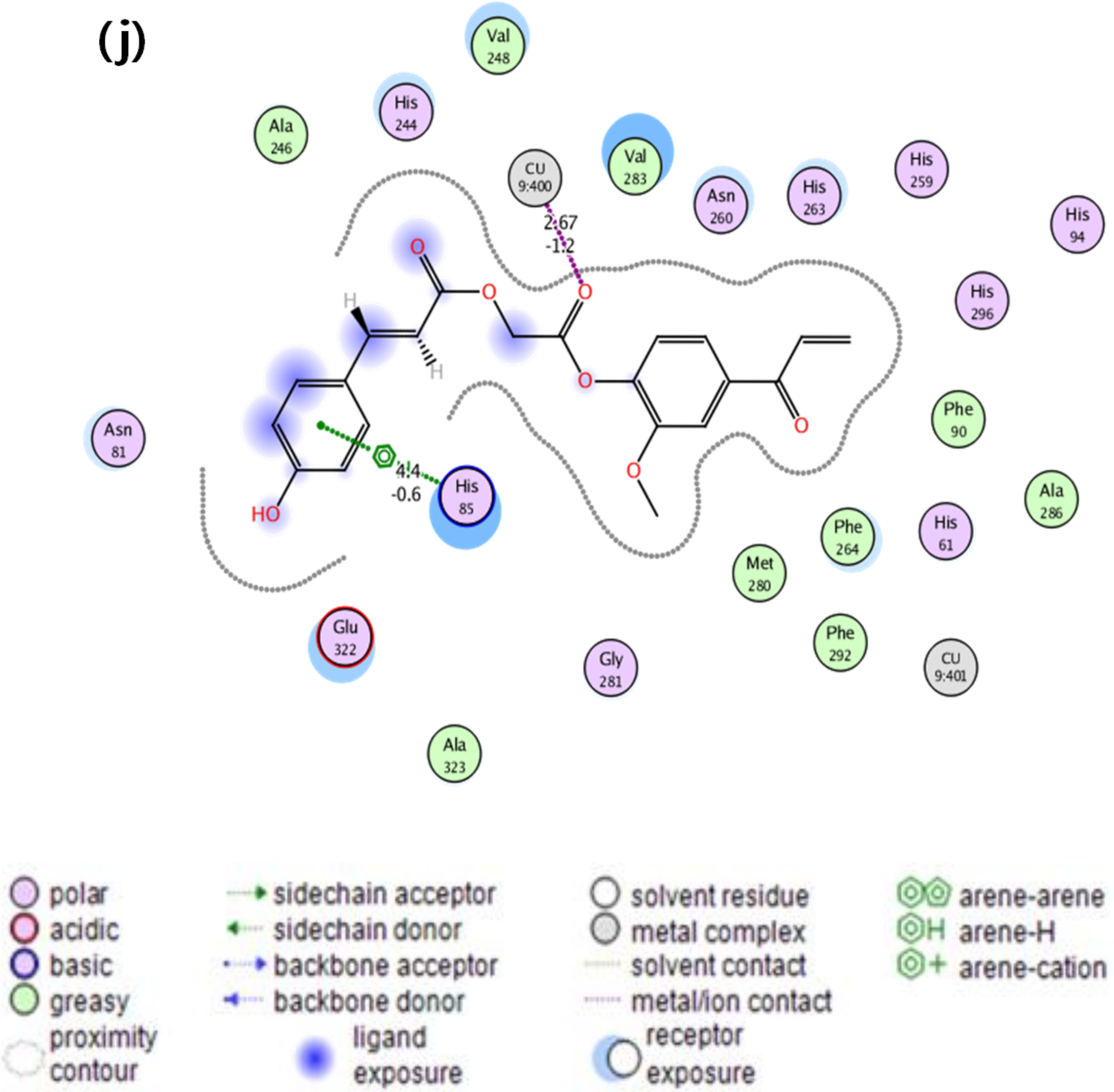
Val 248
His 244
Ala 246
CU 9:400
Val 283
Asn 260
His 263
His 259
His 94
2.67
-1.2
O
H
O
O
H
His 296
O
O
Phe 90
Asn 81
4.4
-0.6
His 85
O
HO
Ala 286
His 61
Phe 264
Met 280
Glu 322
Gly 281
Phe 292
CU 9:401
Ala 323
polar
acidic
basic
greasy
proximity contour
sidechain acceptor
sidechain donor
backbone acceptor
backbone donor
ligand exposure
solvent residue
metal complex
solvent contact
metal/ion contact
receptor exposure
arene-arene
arene-H
arene-cation

Figure S3

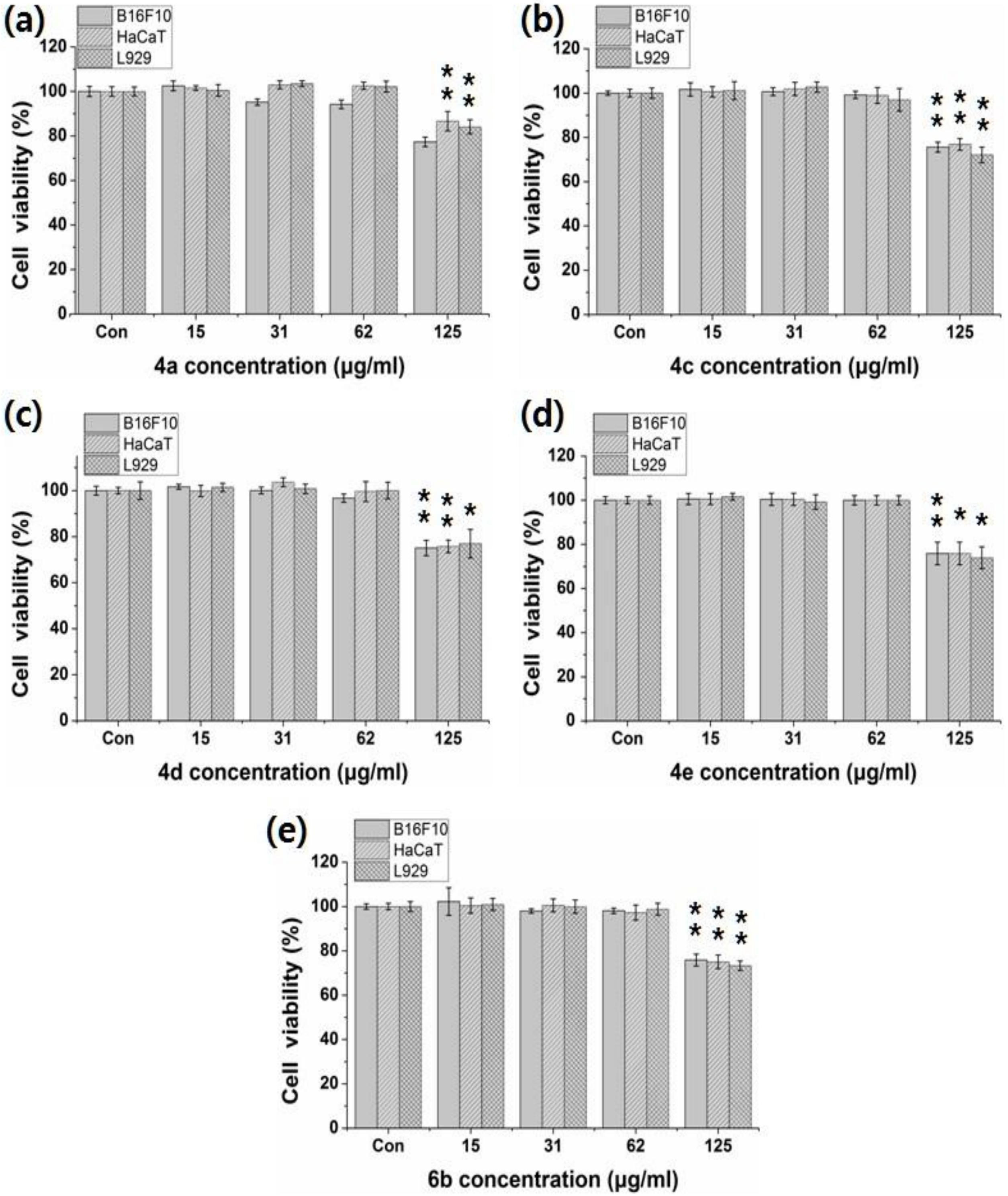

(a)
B16F10
HaCaT
L929
Cell viability (%)
0
20
40
60
80
100
120
Con
15
31
62
125
4a concentration (μg/ml)
(b)
4c concentration (μg/ml)
(c)
4d concentration (μg/ml)
(d)
4e concentration (μg/ml)
(e)
6b concentration (μg/ml)

Figure S4

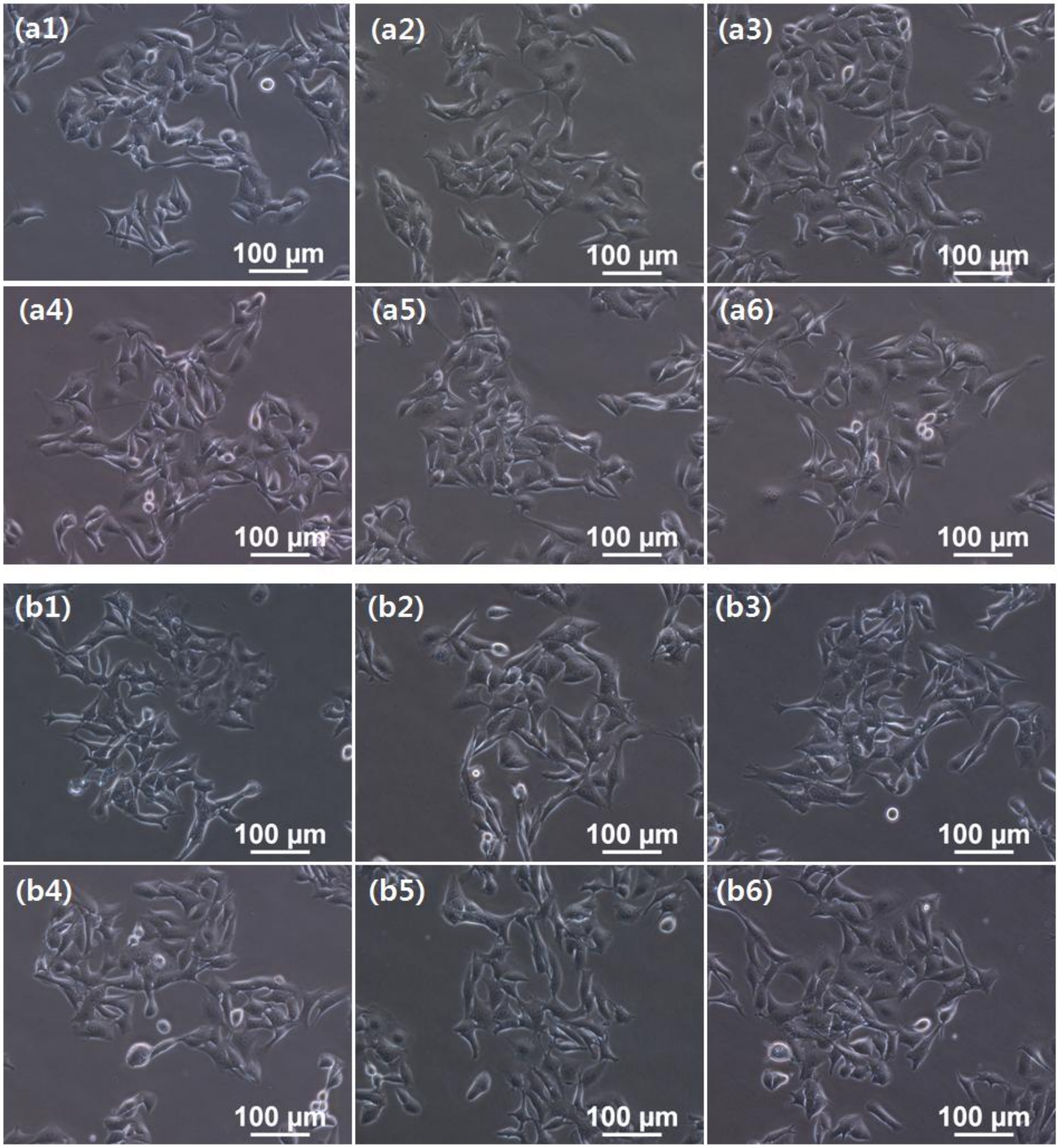
(a1)
100 µm
(a2)
100 µm
(a3)
100 µm
(a4)
100 µm
(a5)
100 µm
(a6)
100 µm
(b1)
100 µm
(b2)
100 µm
(b3)
100 µm
(b4)
100 µm
(b5)
100 µm
(b6)
100 µm

Figure S5

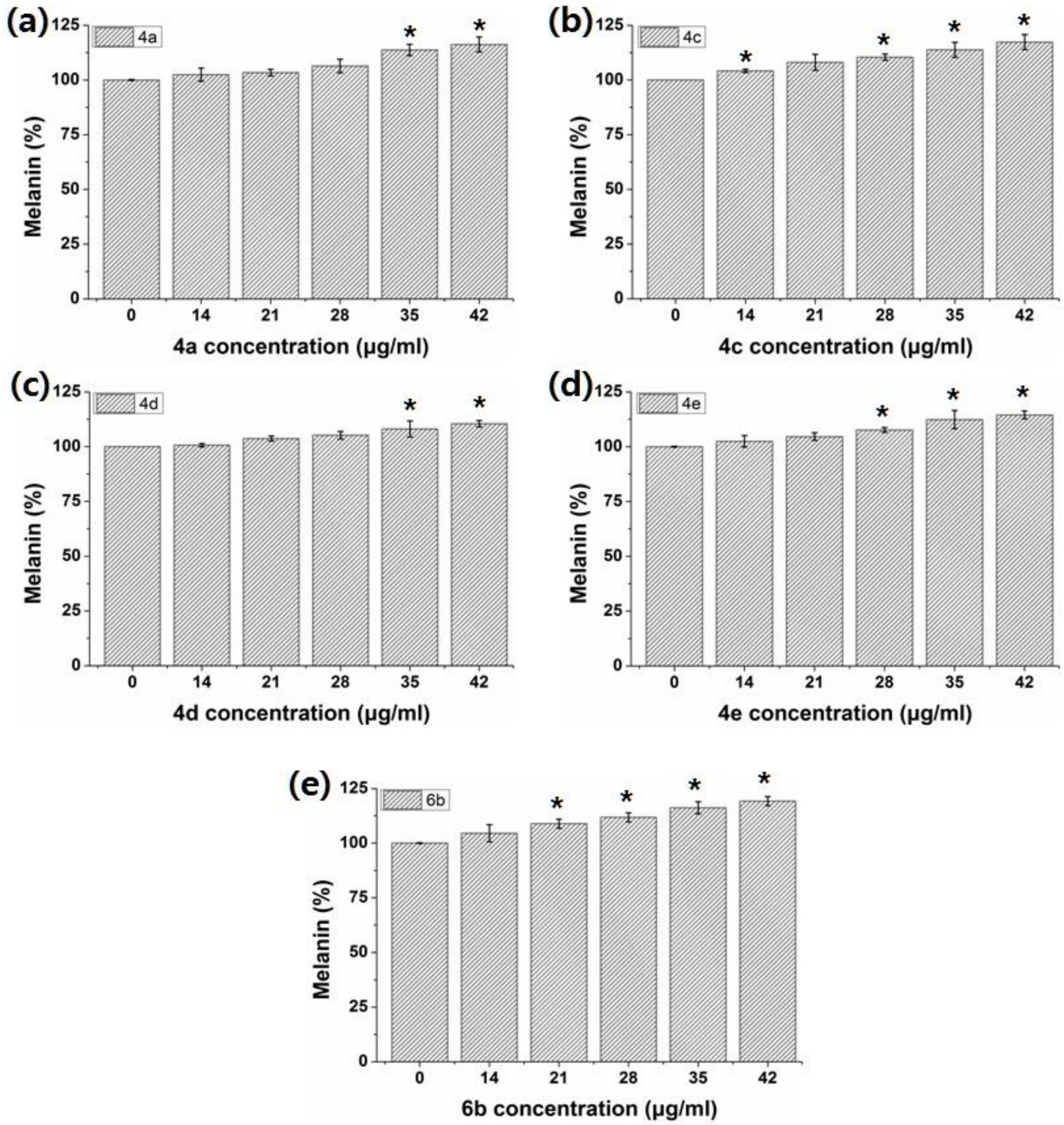

(a)
4a
Melanin (%)
4a concentration (μg/ml)
(b)
4c
Melanin (%)
4c concentration (μg/ml)
(c)
4d
Melanin (%)
4d concentration (μg/ml)
(d)
4e
Melanin (%)
4e concentration (μg/ml)
(e)
6b
Melanin (%)
6b concentration (μg/ml)

Figure S6

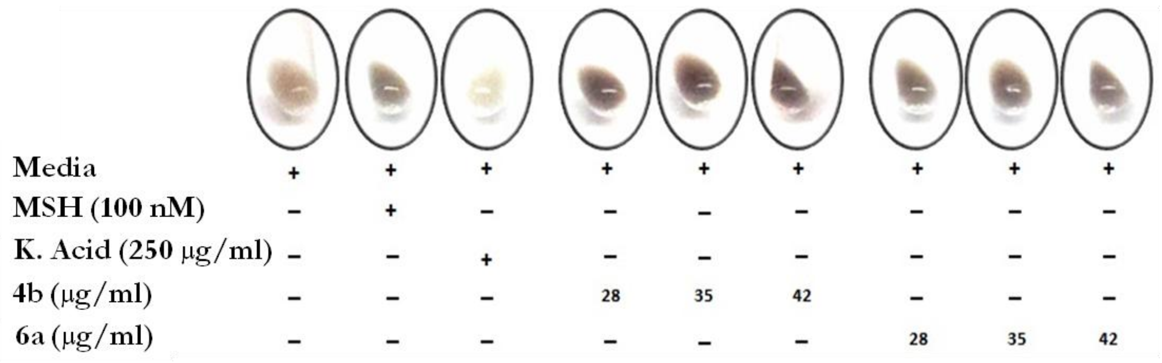

Figure S7
